# Total Synthesis of Resvebassianol A, a Metabolite of Resveratrol by *Beauveria bassiana*

**DOI:** 10.3390/antiox10101509

**Published:** 2021-09-23

**Authors:** Om Darlami, Dongyun Shin

**Affiliations:** College of Pharmacy, Gachon University, 191 Hambakmoe-ro, Yeonsu-gu, Incheon 21936, Korea; darlami.om@gmail.com

**Keywords:** resveratrol, resvebassianol A, *Beauveria bassiana*, metabolites, glycoslyation

## Abstract

Resveratrol is a well-known dietary polyphenol because it has a variety of beneficial biological activities. The fungus *Beauveria bassiana* is one of the most frequently used microorganisms for the biotransformation of polyphenols. Recently, resvebassianol A (**2**), a glycosylated metabolite of resveratrol by *B. bassiana*, was isolated and structurally elucidated. It was demonstrated to exhibit antioxidant, regenerative, and anti-inflammatory activities with no cytotoxicity. Here, we report the first total synthesis of resvebassianol A, 4′-*O*-*β*-(4‴-*O*-methylglucopyranosyl)resveratrol (**2**), and its regiomer, 3-*O*-*β*-(4‴-*O*-methylglucopyranosyl)resveratrol (**3**). Key reactions include (i) the construction of a stilbene core via a novel Heck reaction of aryl halides and styrenes, and (ii) glycosylation with unnatural methylglucopyranosyl bromide. The glycosylation step was carefully optimized by varying the bases and solvents. Resveratrol metabolites **2** and **3** were obtained at 7.5% and 6.3% of the overall yield, respectively.

## 1. Introduction

Resveratrol (**1**, *trans*-3,5,4-trihydroxystilbene) is an important dietary polyphenol and naturally occurring phytoalexin found in grapes, red wine, berries, peanuts, olive oil, etc. [1,2,3]. It is produced by plants in response to environmental stress and fungal attack through the induction of resveratrol synthetase [4,5]. Resveratrol was first isolated from the roots of the white hellebore lily (*Veratrum grandiflorum O. Loes*) in 1940 [6]. Most of the biological activities of resveratrol have been shown by its *trans* stilbene isomer, while the *cis* stilbene isomer also occurs naturally [7]. Resveratrol exerts numerous biological activities such as antioxidant, anti-infective, anti-inflammatory, anti-ischemic, cardioprotective, neuroprotective, anti-aging, anti-viral, anti-obesity, and anti-cancer effects [8,9,10,11,12,13,14,15,16,17,18]. Recently, it was revealed that its ability to activate various deacetylase enzymes (sirtuins) could be responsible for the various biological properties and delay aging [19,20].

Despite their pharmacological activities, various in vivo studies have shown that the potential of polyphenols is impaired by their insolubility in water, ultraviolet light instability, poor intestinal absorption, short half-life, rapid clearance, low bioavailability, and rapid metabolism [21,22]. The introduction of a glycosyl moiety on polyphenols not only helps to enhance the solubility of substrates but also reduces their toxicity, which ultimately increases the activity of biosynthetic intermediates [23]. Moreover, the sugar moiety of polyphenol glycosides might play a major role in their absorption, resulting in an acceptable concentration in the circulatory streams [24]. Polyphenols are subjected to enzymatic oxidation by polyphenol oxidases in plants, during food processing, and also after human consumption, which can be protected by glycosylation [25]. The incorporation of sugar moieties into different types of pharmacophores, natural products, or prodrugs has been proven to improve anti-cancer activities [26].

Several glycosyl derivatives of resveratrol have been recognized in the roots of *Poligonum cuspidatum* such as piceid (3-*O*-*β*-D-glucosyl resveratrol), resveratroloside (4′-*O*-*β*-D-glucosylresveratrol), and 4′-*O*-*β*-D-glucosyl piceatannol [27]. Piceid has been shown to exhibit a broad range of biological activities [28].

**Figure 1:**
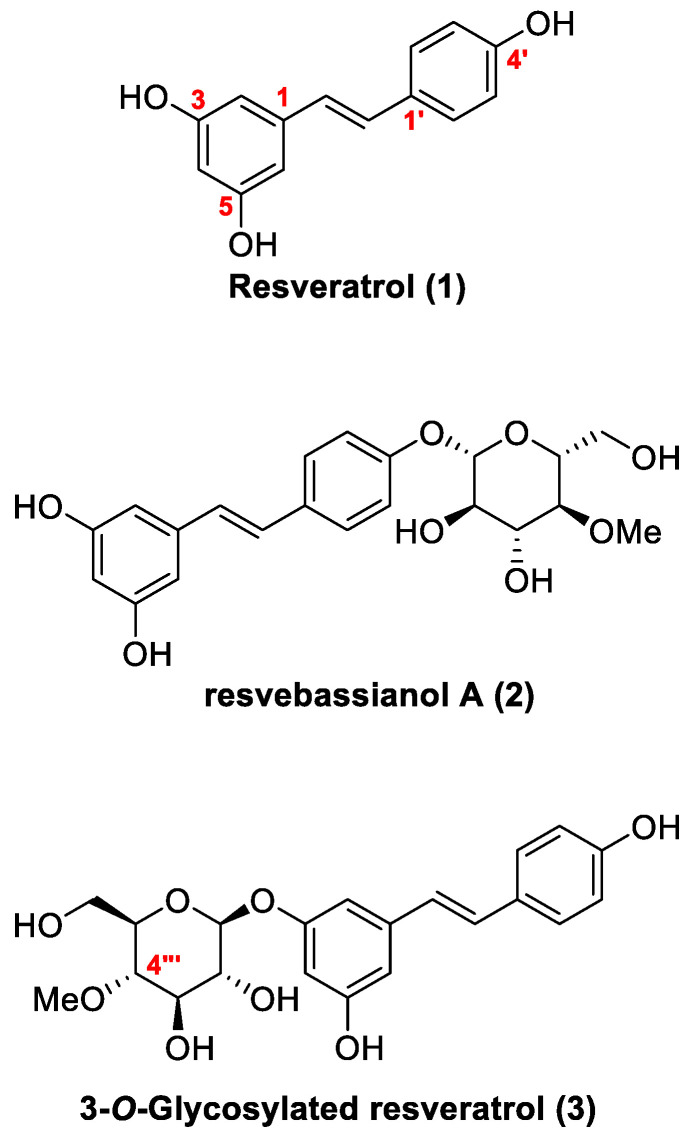
Chemical structures of resveratrol **1**, resvebassianol A (**2**), and 3-*O*-*β*-(4‴-*O*-methylglucopyranosyl) resveratrol **3**.

The fungus *Beauveria bassiana* is the most frequently used biocatalyst and has been used to transform more than 300 bioactive compounds [29,30]. For instance, *B. bassiana* ATCC 7159 has been used for the biotransformation of curvularin and kaempferol, leading to the production of new metabolites resulting from 4-*O*-methyl glucosylation of the substrate, and was highly selective among different hydroxyl groups in the same molecule [30]. Recently, resvebassianol A (**2**) shown in Figure 1, was identified through biotransformation of resveratrol by *B. bassiana* and exhibited important pharmacological activities such as inhibition of inflammatory cytokine expression and cell rejuvenation. Moreover, compared with resveratrol, resvebassianol A proved to be less toxic and more stable [31].

Several synthetic approaches for the formation of glycosidic bonds to phenolic OH in resveratrol have been reported. Direct coupling of resveratrol with a bromo-glucuronide donor was performed by Wang et al. for the synthesis of two glycoconjugates [32]. Coupling of resveratrol with glucuronyl bromide was performed using silver carbonate as an activator, in order to produce glucuronide-conjugated resveratrol in low yield, possibly due to the low solubility of resveratrol in organic solvents. Lucas et al. synthesized resveratrol 3-*O*-*β*-D-glucuronide by coupling a trichloroacetimidate glycosyl donor with protected resveratrol using TMSOTf and BF_3_.OEt_2_ as promoters [3]. Learmonth also synthesized two glucuronide conjugates of resveratrol, in which palladium-catalyzed Heck coupling of an iodo-*O*-*β*-D-glucuronate derivative and its corresponding styrene was adopted [33].

The structural uniqueness and natural resource scarcity of resvebassianol A for biological evaluation prompted us to develop an efficient synthetic method for the metabolite. In this study, we report the total synthesis of resvebassianol A (**2**), a metabolite of resveratrol by *B. bassiana*, and its regiomer, 3-*O*-*β*-(4‴-*O*-methylglucopyranosyl)resveratrol (**3**).

## 2. Materials and Methods

### 2.1. Chemical Reagents

All chemicals and solvents were reagent grade and were purchased from Sigma Aldrich (Saint Louis, MO, USA), TCI (Tokyo, Japan), and Alfa Aesar (Haverhill, MA, USA). All reagents were used directly without further purification.

### 2.2. Purification and Instrumentation

All reactions were carried out in an inert atmosphere in flame-dried glassware. Reactions were monitored by thin-layer chromatography using 0.25 mm silica gel plates and visualized using UV 254/286 nm. Flash chromatography was carried out using silica gel 60 (230–400 mesh, Merck, Darmstadt, Germany) as the stationary phase. ^1^H and ^13^C NMR spectra were recorded using a 600 MHz NMR spectrometer (Bruker, Billerica, MA, USA) with deuterochloroform (CDCl_3_), methanol-d4 (CD_3_OD), or DMSO-d6 (CD_3_)_2_SO. Data for 1H NMR spectra are reported as chemical shifts (multiplicity, coupling constants, integration), and multiplicities are reported as s = singlet, d = doublet, t = triplet, q = quartet, septet = septet, m = multiplet and/or multiple resonances, number of protons, and coupling constant (*J*). High-resolution mass spectra (HRMS) were recorded using electrospray ionization (ESI) mass spectroscopy on a JEOL JMS- 700 (FAB and EI) and an Agilent 6530 Q-TOF LC/MS/MS system (ESI).

### 2.3. General Experimental Procedure

#### 2.3.1. Synthesis of Methyl 4, 6-O-Benzylidene-α-D-Glucopyranoside (**13**)

Methyl *α*-D-glucopyranoside **8** (10 g, 51.5 mmol) was dissolved in anhydrous *N,N*-dimethylformamide (100 mL) under a N_2_ atmosphere, *p*-toluene sulfonic acid (1.62 g, 9.4 mmol) was added, followed by the addition of benzaldehyde dimethyl acetal (9.2 mL, 61.8 mmol), and the solution was stirred under N_2_ for 16 h. After completion of the reaction, triethylamine (4 mL) was added to the reaction, which was then diluted with ethyl acetate. The organic layer was subsequently washed with saturated sodium bicarbonate and brine, dried over Na_2_SO_4_, and concentrated in vacuo. The residue was purified by silica gel column chromatography (methanol/dichloromethane = 30:1) to yield product **13** (12 g, 93%) as a white solid. ^1^H NMR (600 MHz, CDCl_3_) δ 7.49–7.47 (m, Ph, 2H), 7.36–7.34 (m, Ph, 3H), 5.49 (s, 1H), 4.70 (d, *J* = 3.9 Hz, 1H), 4.25 (dd, *J* = 6, 6 Hz, 1H), 3.87 (t, *J* = 9 Hz, 1H), 3.76–3.74 (m, 1H), 3.69 (t, *J* = 12, 1H), 3.57–3.53 (m, 2H), 3.43 (t, *J* = 12 Hz, 1H), 3.39 (s, 3H), 2.93 (d, *J* = 6 Hz, 1H); ^13^C NMR (151 MHz, CDCl_3_) δ 137.0, 129.2, 128.3, 126.4, 101.9, 99.9, 80.9, 72.7, 71.4, 68.9, 62.3, 55.5; HRMS (ESI): mass calcd for C_14_H_18_O_6_ [M + H]^+^, 283.1176; found, 283.1168.

#### 2.3.2. Synthesis of Methyl 2,3-di-O-Benzyl-4,6-O-Benzylidene-α-D-Glucopyranoside (**14**)

Methyl 4,6-*O*-benzylidene-α-D-glucopyranoside **13** (11.5 g, 40.73 mmol) was dissolved in anhydrous *N*,*N*-dimethylformamide (100 mL) under a N_2_ atmosphere. The solution was cooled to 0 °C in an ice bath, after which NaH (60% dispersion in mineral oil, 4 g, 163 mmol) was added, and the reaction was stirred for 1 h at room temperature. The solution was cooled to 0 °C, and benzylbromide (14.5 mL, 122 mmol) was added dropwise. The solution was stirred at room temperature overnight, after which methanol (10 mL) was added, and the mixture was concentrated under reduced pressure. The residue was dissolved in CH_2_Cl_2_ (200 mL), washed with water (2 × 75 mL) and brine (1 × 75 mL), and dried over MgSO_4_. The residue was purified by silica gel column chromatography (ethyl acetate/n-hexane = 1:7) to yield product **14** (17.5 g, 92%) as a white solid compound. ^1^H NMR (600 MHz, CDCl_3_) δ 7.49 (d, *J* = 6, 2H), 7.39–7.21 (m, 13H), 5.54 (s, 1H), 4.91 (d, *J* = 12 Hz, 1H), 4.84 (dd, *J* = 12 Hz, 2H), 4.70 (d, *J* = 12 Hz, 1H), 4.60 (d, *J* = 6 Hz, 1H), 4.26 (dd, *J* = 12, 6 Hz, 1H), 4.05 (t, *J* = 9 Hz, 1H), 3.84–3.80 (m, 1H), 3.70 (t, *J* = 9 Hz, 1H), 3.60 (t, *J* = 9Hz, 1H), 3.55 (dd, *J* = 3 Hz, 1H), 3.39 (s, 3H); ^13^C NMR (151 MHz, CDCl_3_) δ 138.8, 138.2, 137.5, 129.0, 128.5, 128.4, 128.3, 128.2, 128.1, 128, 127.7, 126.1, 101.3, 99.3, 82.2, 79.2, 78.6, 75.4, 73.8, 69.1, 62.4, 55.4; HRMS (ESI): mass calcd for C_28_H_30_O_6_ [M + H]^+^, 463.2115; found, 463.2112.

#### 2.3.3. Synthesis of Methyl 2,3,6-tri-O-Benzyl-α-D-Glucopyranoside (**15**)

Methyl 2,3-di-*O*-benzyl-4,6-*O*-benzylidene-*α*-D-glucopyranoside **14** (17.25 g, 38 mmol) was dissolved in anhydrous CH_2_Cl_2_ (100 mL) under a N_2_ atmosphere, and the solution was cooled to 0 °C. Et_3_SiH (30 mL, 186 mmol) and trifluoroacetic acid (14 mL, 186 mmol) were added, and the solution was stirred at 0 °C for 4 h. The reaction was quenched with Et_3_N and methanol. CH_2_Cl_2_ was added, and the solution was washed with water and brine, dried over MgSO_4_, filtered, and concentrated under reduced pressure. The mixture was purified by silica gel column chromatography (ethyl acetate/n-hexane = 1:4) to afford product **15** (12 g, 91%) as a colorless oil. ^1^H NMR (600 MHz, CDCl_3_) δ 7.37–7.26 (m, 15H), 5 (d, *J* = 12 Hz, 1H), 4.76 (dd, *J* = 12 Hz, 2H), 4.76–4.63 (m, 2H), 4.59 (d, *J* = 12 Hz, 1H), 4.54 (d, *J* = 12, 1H), 3.79 (t, *J* = 12, 18 Hz, 1H), 3.71–3.68 (m, 3H), 3.61 (t, *J* = 12, 18 Hz, 1H), 3.54 (dd, *J* = 6 Hz, 1H), 3.38 (s, 3H), 2.33 (s, 1H); ^13^C NMR (151 MHz, CDCl_3_) δ 138.8, 138.1, 138.0, 128.6, 128.5, 128.4, 128.1, 128.0, 128.0, 127.9, 127.7, 127.6, 98.2, 81.5, 79.6, 77.2, 77.0, 76.8, 75.4, 73.6, 73.2, 70.7, 69.9, 69.5, 55.3; HRMS (ESI): mass calcd for C_28_H_32_O_6_ [M + NH_4_]^+^, 482.2537; found, 487.2525.

#### 2.3.4. Synthesis of Methyl 2,3,6-Tri-O-Benzyloxy-4-O-Methyl-α-D-Glucopyranoside (**16**)

NaH (60% dispersion in mineral oil, 1.5 g, 63 mmol) was added to a solution of methyl 2,3,6-tri-*O*-benzyloxy-*α*-D-glucopyranoside **15** (11.5 g, 25 mmol) in anhydrous *N,N*- dimethylformamide (100 mL) at 0 °C. The reaction mixture was stirred for 1 h at 0 °C, and methyl iodide (3.8 mL, 63 mmol) was added to the reaction mixture. The reaction mixture was stirred overnight at room temperature. The reaction was quenched with methanol and ice-cold water and then extracted with ethyl acetate. The collected organic layers were washed with brine, dried with Na_2_SO_4_, and concentrated under reduced pressure. The mixture was purified by silica gel column chromatography (ethyl acetate/n-hexane = 1:7) to yield product **16** (11.2 g, 95%) as a viscous liquid. ^1^H NMR (600 MHz, CDCl_3_) δ 7.39–7.26 (m, 5H), 4.93 (d, *J* = 10.8, 1H), 4.83–4.74 (m, 2H), 4.67–4.57 (m, 3H), 4.51 (d, *J* = 12.1 Hz, 1H), 3.86 (s, 1H), 3.66 (dd, *J* = 22.7, 7.1 Hz, 3H), 3.50 (dd, *J* = 9.7, 3.5 Hz, 1H), 3.46 (s, 3H), 3.37 (s, 3H), 3.33 (s, 1H); ^13^C NMR (151 MHz, CDCl_3_) δ 138.9, 138.2, 138.0, 128.4, 128.4, 128.3, 128.1, 128.0, 127.9, 127.8, 127.6, 127.6, 98.2, 82.1, 79.6, 79.4, 75.7, 73.5, 73.4, 70.1, 68.6, 60.7, 55.2; HRMS (ESI): mass calcd for C_29_H_34_O_6_ [M + NH_4_]^+^, 496.2694; found, 496.2688.

#### 2.3.5. Synthesis of Methyl 4-O-Methyl-α-D-Glucopyranoside (**17**)

Pd (10%)/C (3 g) was added to a solution of methyl 2,3,6-Tri-*O*-benzyloxy-4-*O*-methyl-*α*-D-glucopyranoside **16** (11 g, 22 mmol) in anhydrous methanol (100 mL), and the mixture was stirred under an atmosphere of hydrogen at room temperature for 24 h. The catalyst was filtered out, and the solvents were removed under reduced pressure. The crude residue was purified by silica gel column chromatography (ethyl acetate/n-hexane = 1:1) to afford the viscous product **17** (4.6 g, 95%). ^1^H NMR (600 MHz, CD_3_OD) δ 4.69 (s, 1H), 3.78 (d, *J* = 11.7 Hz, 1H), 3.70 (dq, *J* = 12.2, 6.9, 4.4 Hz, 2H), 3.57 (s, 3H), 3.53–3.47 (m, 1H), 3.44 (d, *J* = 7.9 Hz, 1H), 3.40 (s, 3H), 3.10 (t, *J* = 9.3 Hz, 1H); ^13^C NMR (151 MHz, CD_3_OD) δ 99.7, 79.6, 73.7, 72.1, 71.2, 60.8, 59.5, 54.2; HRMS (ESI): mass calcd for C_8_H_16_O_6_ [M + H]^+^, 209.1020; found, 209.1033.

#### 2.3.6. Synthesis of Methyl 2,3,6-tri-O-Acetyl-α-D-Glucopyranoside (**18**)

Compound **17** (4.5 g, 22 mmol) was dissolved in acetic anhydride (25 mL, 217 mmol) and pyridine (25 mL, 217 mmol) and stirred at room temperature for 12 h. After completion of the reaction, pyridine and acetic anhydride were removed in vacuo. The residue was dissolved in CH_2_Cl_2_ and washed with dilute HCl. The organic layer was collected, washed with brine, dried with MgSO_4_, and concentrated under reduced pressure. The mixture was purified by silica gel column chromatography (ethyl acetate/n-hexane = 1:4) to afford the viscous product **18** (5.1 g, 71%). ^1^H NMR (600 MHz, CDCl_3_) δ 5.50–5.42 (m, 1H), 4.88–4.79 (m, 2H), 4.35 (d, *J* = 10.0 Hz, 1H), 4.30–4.24 (m, 1H), 3.88–3.81 (m, 1H), 3.42 (d, *J* = 3.7 Hz, 3H), 3.39 (d, *J* = 3.9 Hz, 3H), 3.34 (dd, *J* = 12.6, 6.3 Hz, 1H), 2.12 (d, *J* = 3.9 Hz, 3H), 2.08 (d, *J* = 6.0 Hz, 6H); ^13^C NMR (150 MHz, CDCl_3_) δ 170.7, 170.4, 169.8, 96.8, 77.8, 71.9, 71.1, 68.2, 62.7, 60.1, 55.3, 20.9, 20.8, 20.8; HRMS (ESI): mass calcd for C_14_H_22_O_9_ [M + NH_4_]^+^, 352.1602; found, 352.1605.

#### 2.3.7. Synthesis of 1,2,3,6-Tetra-O-Acetyl-4-O-Methyl-α-D-Glucopyranoside (**19**)

To a stirred solution of **18** (5 g, 15 mmol) in acetic anhydride (50 mL) at 0 °C, boron trifluoride ether (2 mL, 15 mmol) was added. The solution was warmed to room temperature and allowed to stir for 2 h. Then, the solution was poured into an ice-cold saturated solution of NaHCO_3_ and extracted with ethyl acetate. The combined organic layers were separated, dried over MgSO_4_, and concentrated under reduced pressure. The mixture was purified by silica gel column chromatography (ethyl acetate/n-hexane = 1:2) to yield the viscous product **19** (4.1 g, 75%). ^1^H NMR (600 MHz, CDCl_3_) δ 6.25 (t, *J* = 3.1 Hz, 1H), 5.45 (ddd, *J* = 10.2, 9.2, 2.4 Hz, 1H), 5.0 (ddd, *J* = 10.3, 3.7, 2.7 Hz, 1H), 4.35–4.31 (m, 1H), 4.28–4.24 (m, 1H), 3.99–3.92 (m, 1H), 3.45 (d, *J* = 2.3 Hz, 3H), 3.43 (d, *J* = 2.2 Hz, 1H), 2.16 (d, *J* = 2.3 Hz, 3H), 2.12–2.09 (m, 6H), 2.01 (d, *J* = 2.4 Hz, 3H); ^13^C NMR (151 MHz, CDCl_3_) δ 170.6, 170.0, 169.9, 169.0, 89.2, 71.7, 71.0, 69.6, 62.3, 60.5, 20.9, 20.8, 20.5; HRMS (ESI): mass calcd for C_15_H_22_O_10_ [M + NH_4_]^+^, 380.1551; found, 380.1545.

#### 2.3.8. Synthesis of 2,3,6-O-Triacetyl-4-O-Methylglucopyranosyl bromide (**6**)

A solution of HBr (10 mL, 33 wt% in acetic acid) was added dropwise to a stirred solution of compound **19** (3.7 g, 10 mmol) in CH_2_Cl_2_ (50 mL) at 0 °C. The solution was stirred at room temperature for 4 h. After completion of the reaction, the reaction mixture was quenched carefully with ice water and diluted with CH_2_Cl_2_ and water. The organic layer was separated and washed with saturated NaHCO_3_ and brine. The organic layer was dried over MgSO_4_, filtered, and concentrated under reduced pressure. The residue was purified by silica gel column chromatography (ethylacetate/n-hexane 1:4) to yield the light yellow liquid **6** (2.8 g, 73%). This compound was unstable, and after drying, it was used for further reactions. ^1^H NMR (600 MHz, CDCl_3_) δ 6.53 (d, 1H, *J* = 3.7 Hz), 5.57 (t, *J* = 9.6 Hz, 1H), 4.75 (dd, *J* = 10.0, 3.8 Hz, 1H), 4.39 (d, *J* = 12.4 Hz, 1H), 4.31 (dd, *J* = 12.4, 4.0 Hz, 1H), 4.15 (d, *J* = 10.1 Hz, 1H), 3.45 (d, *J* = 9.7 Hz, 4H), 2.12 (d, *J* = 6.6 Hz, 6H), 2.10 (s, 3H); ^13^C NMR (151 MHz, CDCl_3_) δ 170.5, 170.1, 169.6, 86.8, 76.6, 73.2, 71.8, 70.9, 61.8, 60.3, 20.9, 20.8, 20.7.

#### 2.3.9. Synthesis of 3,5-bis(tert-Butyldimethylsilyloxy) Benzaldehyde (**20**)

To a well-stirred solution of 3,5-dihydroxybenzaldehyde **9** (2 g, 14.48 mmol) and DIPEA (5.3 mL, 43.4 mmol) in *N,N*- dimethylformamide (20 mL), *tert*-butylchlorodimethylsilane (6.55 g, 43.4 mmol) was added at 0 °C, and the reaction mixture was stirred for 3 h at room temperature. The reaction mixture was diluted with CH_2_Cl_2_ and washed with saturated aqueous NaCl, and the combined organic layers were dried over anhydrous Na_2_SO_4_ and concentrated under reduced pressure. The residue was purified by silica gel column chromatography (ethyl acetate/n-hexane = 1:20) to afford **20** (5.1 g, 96%) as a colorless oil. ^1^H NMR (600 MHz, CDCl_3_) δ 9.9 (s, 1H), 7.0 (s, 2H), 6.6 (s, 1H), 1.0 (s, 18H), 0.2 (s, 12H); ^13^C NMR (151 MHz, CDCl_3_) δ 191.9, 157.3, 138.3, 118.4, 114.4, 25.7, 25.6, 18.2; HRMS (ESI): mass calcd for C_19_H_34_O_3_Si_2_[M + H]^+^, 367.2119; found, 367.2113.

#### 2.3.10. Synthesis of (5-Vinyl-1,3-Phenylene)bis(oxy)bis(tert-Butyldimethylsilane (**21**)

A mixture of methyltriphenylphosphonium bromide (7.3 g, 20.4 mmol) and potassium *tert*-butoxide (2.2 g, 20 mmol) in anhydrous THF was refluxed for 1 h for in situ generation of methylenetriphenylphosphorane. Upon returning to room temperature, a solution of 3,5-di(*tert*-butyldimethylsilyloxy) benzaldehyde **20** (5 g, 13.6 mmol) in anhydrous THF was added dropwise, and the reaction was heated to reflux overnight. After completion of the reaction, ethyl acetate was added, and the solution was washed with water, dried over anhydrous Na_2_SO_4_, filtered, and concentrated under reduced pressure. The residue was purified by silica gel column chromatography (ethyl acetate/n-hexane = 1:25) to yield product **21** (3.4 g, 68%) as a colorless oil. ^1^H NMR (600 MHz, CDCl_3_) δ 6.6 (dd, *J* = 17.4, 10.9 Hz, 1H), 6.5 (s, 2H), 6.3 (s, 1H), 5.7 (d, *J* = 17.5 Hz, 1H), 5.2 (d, *J* = 10.9 Hz, 1H), 1.0 (s, 18H), 0.2 (s, 12H); ^13^C NMR (151 MHz, CDCl_3_) δ 156.6, 139.4, 136.7, 113.9, 111.7, 111.5, 25.7, 25.7, 18.2; HRMS (ESI): mass calcd for C_20_H_36_O_2_Si_2_[M + H]^+^, 365.2327; found, 365.2333.

#### 2.3.11. Synthesis of 5-Vinylbenzene-1,3-diol, [3,5-Dihdroxy Styrene] (**12**)

To a solution of (5-vinyl-1,3-phenylene) bis(oxy)bis(tert-butyldimethylsilane) **21** (3 g, 8.2 mmol) in anhydrous THF (15 mL), TBAF (4 mL, 14 mmol) was added at 0 °C, and the reaction mixture was stirred for 3 h at room temperature. The volume was reduced by rotary evaporation, and ethyl acetate was added. The organic layer was washed with water, dried over anhydrous Na_2_SO_4_, filtered, and concentrated under reduced pressure. The residue was purified by silica gel column chromatography (ethyl acetate/n-hexane = 1:1) to yield product **12** (1.2 g, 92%) as a viscous pale oil. ^1^H NMR (600 MHz, CD_3_OD) δ 6.6 (dd, *J* = 17.4, 11.0 Hz, 1H), 6.4 (s, 2H), 6.2 (s, 1H), 5.6 (d, *J* = 17.6 Hz, 1H), 5.1 (d, *J* = 10.8 Hz, 1H); ^13^C NMR (151 MHz, CD_3_OD) δ 158.2, 139.6, 137.0, 112.3, 104.4, 101.8, 35.6, 35.2; HRMS (ESI): mass calcd for C_8_H_8_O_2_[M + H]^+^, 137.0597; found, 137.0621.

#### 2.3.12. Synthesis of 5-Vinyl-1,3-Phenylene Diacetate (3,5-Diacetoxystyrene) (**5**)

Acetic anhydride (2 mL, 22 mmol) was added dropwise to a solution of compound **12** (1 g, 7.3 mmol), pyridine (1.9 mL, 22 mmol), and DMAP (26 mg, 0.219 mmol) in CH_2_Cl_2_ at 0 °C. The reaction mixture was stirred at room temperature for 12 h. The solution was concentrated under reduced pressure, and the residue was purified by silica gel column chromatography (ethyl acetate/n-hexane = 1:4) to yield 5-vinyl-1,3-phenylene diacetate **5** (1.12 g, 70%) as a clear oil. ^1^H NMR (600 MHz, CDCl_3_) δ 7.0 (s, 2H), 6.8 (s, 1H), 6.6 (dd, 1H, *J* = 17.1, 11.2 Hz), 5.7 (d, 1H, *J* = 17.5 Hz), 5.3 (d, 1H, *J* = 10.8 Hz), 2.3 (s, 6H); ^13^C NMR (151 MHz, CDCl_3_) δ 169.0, 151.2, 139.9, 135.3, 116.7, 115.9, 114.7, 21.0; HRMS (ESI): mass calcd for C_12_H_12_O_4_[M + NH_4_]^+^, 238.1074; found, 238.1085.

#### 2.3.13. Synthesis of 4-Iodophyenyl Acetate (**11**)

To a well-stirred mixture of 4- iodophenol **7** (2 g, 9 mmol) in dry pyridine (6 mL), acetic anhydride (1.75 mL, 18 mmol) was added at room temperature under N_2_. The mixture was then stirred for 12 h. After completion of the reaction, water was added and extracted with CH_2_Cl_2_. The organic layer was dried over MgSO_4_ and concentrated under reduced pressure. The residue was purified by silica gel column chromatography (dichloromethane 100%) to yield product **11** (2.2 g, 95%) as a white solid. ^1^H NMR (600 MHz, CDCl_3_) δ 7.7–7.6 (m, 2H), 6.9–6.8 (m, 2H), 2.3 (s, 3H); ^13^C NMR (151 MHz, CDCl_3_) δ 169.1, 150.5, 138.5, 123.8, 89.9, 21.1; HRMS (ESI): mass calcd for C_8_H_7_ IO_2_[M + NH_4_]^+^, 279.9827; found, 279.9826.

#### 2.3.14. Synthesis of 4-Iodophenyl-2′,3′,6′-O-triacetyl-4′-O-Methylglucopyranoside (**4**)

To a mixture of iodophenol **7** (259 mg, 1.9 mmol) and 2,3,6-*O*-triacetyl-4-methylglucopyranosyl bromide **6** (730 mg, 1.9 mmol) in CHCl_3_, benzyltributylammonium chloride (60 mg, 0.19 mmol) and potassium carbonate (665 mg, 4.8 mmol) were added and stirred at room temperature for 24 h. The reaction mixture was neutralized with 1 N HCl, and the organic layer was separated. The organic layer was washed with water-saturated NaHCO_3_ and brine, dried over MgSO_4_, and concentrated under reduced pressure. The residue was purified by silica gel column chromatography (ethyl acetate/n-hexane = 1:4) to yield product **4** (565 mg, 57%) as a white solid. ^1^H NMR (600 MHz, CDCl_3_) δ 7.56–7.54 (m, 2H), 6.74 (dd, *J* = 8.9, 2.7 Hz 2H), 5.22 (t, *J* = 9.3 Hz, 1H), 5.12 (dd, *J* = 9.6, 7.7 Hz, 1H), 4.98 (d, *J* = 7.8 Hz, 1H), 4.38 (dd, *J* = 12.0, 2.4 Hz, 1H), 4.24 (dd, *J* = 12.1, 5.6 Hz, 1H), 3.67 (ddd, *J* = 10.1, 5.7, 2.4 Hz, 1H), 3.43 (d, *J* = 2.5 Hz, 4H), 2.08 (d, *J* = 8.5 Hz, 6H), 2.03 (d, *J* = 2.5 Hz, 3H); ^13^C NMR (151 MHz, CDCl_3_) δ 170.5, 170.0, 169.6, 156.7, 138.4, 119.2, 98.7, 86.0, 77.5, 74.7, 73.1, 71.5, 62.7, 60.5, 20.9, 20.8, 20.7; HRMS (ESI): mass calcd for C_19_H_23_IO_9_[M + NH_4_]^+^, 540.0725; found, 540.0712.

#### 2.3.15. Synthesis of (E)-1″-(3,5-Diacetoxy)-2″-(4′-O-2‴,3‴,6‴-Triacetyl-4‴-O-Methyl-β-D Glucopyranosidophenyl) Ethene (**22**)

To a solution of 3,5-diacetoxystyrene **5** (235 mg, 1.07 mmol) and compound **4** (560 mg, 1.07 mmol) in acetonitrile, palladium(II) acetate (0.012 mg, 0.053 mmol), benzyltriethylammonium chloride (243 mg,1.07 mmol), and tributylamine (0.68 mL, 2.9 mmol) were added and stirred at 100 °C for 2 h, N_2_. After 2 h, the mixture was cooled to room temperature, filtered through a short Celite pad, and then evaporated to dryness. The residue was taken up in dichloromethane, washed with diluted HCl, water, and brine, dried over anhydrous MgSO_4_, filtered, and concentrated under reduced pressure. The residue was purified by silica gel column chromatography (ethyl acetate/n-hexane = 1:2) to yield compound **22** (361 mg, 55%) as a white crystal. ^1^H NMR (600 MHz, CDCl_3_) δ 7.41 (d, *J* = 8.5 Hz, 2H), 7.10 (s, 2H), 7.02 (d, *J* = 16.2 Hz, 1H), 6.97 (d, *J* = 8.5 Hz, 2H), 6.91 (d, *J* = 16.2 Hz, 1H), 6.80 (s, 1H), 5.26 (t, *J* = 9.3 Hz, 1H), 5.17 (t, *J* = 8.7 Hz, 1H), 5.07 (d, *J* = 7.8 Hz, 1H), 4.42 (d, *J* = 11.9 Hz, 1H), 4.29 (dd, *J* = 11.9, 5.4 Hz, 1H), 3.74–3.68 (m, 1H), 3.46 (s, 4H), 2.30 (s, 6H), 2.11 (d, *J* = 5.5 Hz, 6H), 2.06 (s, 3H). ^13^C NMR (151 MHz, CDCl_3_) δ 170.6, 170.1, 169.7, 169.0, 156.8, 151.3, 139.7, 131.9, 129.7, 127.9, 126.0, 117.1, 116.8, 114.2, 98.7, 77.6, 74.8, 73.1, 71.6, 62.7, 60.5, 21.1, 20.9, 20.8, 20.7; HRMS (ESI): mass calcd for C_31_H_34_O_13_[M + NH_4_] ^+^, 632.2338; found, 632.2342.

#### 2.3.16. Synthesis of 4′-O-β- (4‴-O-Methylglucopyranosyl)Resveratrol (**2**)

Compound **22** (350 mg, 0.58 mmol) was dissolved in methanol (20 mL) and 0.2 M methanolic solution of sodium methoxide (20 mL). The resulting mixture was stirred for 1 h at room temperature. After completion of the reaction, the mixture was concentrated under reduced pressure. The residue was purified by silica gel column chromatography (methanol/dichloromethane = 1:8) to obtain the final compound **2** (195 mg, 85%) as a white powder. ^1^H NMR (600 MHz, (CD_3_)_2_CO) δ 8.23 (s, 2H), 7.50 (d, *J* = 8.3 Hz, 2H), 7.04 (d, *J* = 7.6 Hz, 3H), 6.97 (s, 1H), 6.56 (s, 2H), 6.28 (s, 1H), 4.96 (d, *J* = 7.7 Hz, 1H), 4.64 (s, 1H), 4.40 (d, *J* = 3.4 Hz, 1H), 3.84 (dd, *J* = 10.8, 4.5 Hz, 1H), 3.80–3.75 (m, 1H), 3.70 (dd, *J* = 11.7, 5.1 Hz, 1H), 3.63 (dd, *J* = 8.8, 3.3 Hz, 1H), 3.57 (s, 3H), 3.51–3.43 (m, 2H), 3.31 (d, *J* = 5.2 Hz, 1H), 3.22 (t, *J* = 9.3 Hz, 1H). ^13^C NMR (151 MHz, (CD_3_)_2_CO) δ 158.7, 157.5, 139.7, 131.5, 127.7, 127.5, 127.3, 116.6, 104.9, 102.0, 100.6, 79.2, 77.1, 76.1, 74.0, 61.2, 59.7; HRMS (ESI): mass calcd for C_21_H_24_O_8_[M + H]^+^, 405.1544; found, 405.1551.

#### 2.3.17. Synthesis of 3-Hydroxy-5-Vinylphenyl-2′,3′,6′-tri-O-Acetyl-4′-O-Methyl-β-D-Glucopyranoside (**10**)

To a solution of dihydroxystyrene **12** (248 mg, 1.8 mmol) and 2,3,6-*O*-triacetyl-4 methylglucopyranosyl bromide **6** (700 mg, 1.8 mmol) in CHCl_3_, benzyltributylammonium chloride (56 mg, 0.18 mmol) and potassium carbonate (636 mg, 4.6 mmol) were added and stirred at room temperature for 24 h. The reaction mixture was neutralized with 1 N HCl, and the organic layer was separated. The organic layer was washed with saturated NaHCO_3_ and brine, dried over MgSO_4_, and concentrated under reduced pressure. The residue was purified by silica gel column chromatography (ethyl acetate/n-hexane = 1:2) to yield product **10** (320 mg, 40%) as a white product. ^1^H NMR (600 MHz, CDCl_3_) δ 6.59 (d, *J* = 17.1 Hz, 3H), 6.42 (s, 1H), 6.04 (s, 1H), 5.69 (d, *J* = 17.5 Hz, 1H), 5.25–5.23 (m, 2H), 5.13 (t, *J* = 8.6 Hz, 1H), 5.02 (d, *J* = 7.7 Hz, 1H), 4.42 (d, *J* = 11.8 Hz, 1H), 4.26 (dd, *J* = 11.7, 5.6 Hz, 1H), 3.71–3.69 (m, 1H), 3.45 (d, *J* = 12.0 Hz, 4H), 2.10 (d, *J* = 10.5 Hz, 6H), 2.05 (s, 3H); ^13^C NMR (151 MHz, CDCl_3_) δ 170.3, 170.0, 158.1, 157.0, 140.0, 136.2, 114.9, 108.2, 107.1, 104.1, 98.7, 77.7, 74.8, 73.0, 71.7, 62.8, 60.5, 20.9, 20.8, 20.7; HRMS (ESI): mass calcd for C_21_H_26_O_10_[M + NH_4_]^+^, 456.1864; found, 456.1875.

#### 2.3.18. Synthesis of 3-Acetoxy-5-Vinylphenyl-2′,3′,6′-tri-O-Acetyl-4′-O-Methyl-β-D-Glucopyranoside (**23**)

Compound **10** (310 mg, 0.7 mmol) in CH_2_Cl_2_ (5 mL) at room temperature was added to pyridine (0.1 mL, 1.06 mmol) and 4-dimethylaminopyridine (0.001 mg), and acetic anhydride (0.1 mL, 1.06 mmol) was added dropwise. The resulting mixture was stirred for 1 h. The mixture was diluted with CH_2_Cl_2_ and water. The organic phase was separated and washed with dilute hydrochloric acid, water, and brine, dried over anhydrous MgSO_4_, filtered, and dried under reduced pressure. The residue was purified by silica gel column chromatography (ethyl acetate/n-hexane = 1:4) to yield product **23** (288 mg, 85%) as a white solid. ^1^H NMR (600 MHz, CDCl_3_) δ 6.86 (dd, *J* = 30.6, 2.0 Hz, 2H), 6.65–6.56 (m, 2H), 5.74–5.68 (m, 1H), 5.31–5.20 (m, 2H), 5.14 (ddt, *J* = 10.5, 7.8, 1.1 Hz, 1H), 5.05 (d, *J* = 7.7 Hz, 1H), 4.40 (dq, *J* = 12.0, 1.6 Hz, 1H), 4.27–4.20 (m, 1H), 3.75–3.68 (m, 1H), 3.47–3.39 (m, 5H), 2.27 (dd, *J* = 2.4, 1.1 Hz, 3H), 2.09 (dd, *J* = 2.3, 1.1 Hz, 3H), 2.07 (dd, *J* = 2.3, 1.1 Hz, 3H), 2.04 (dd, *J* = 2.3, 1.1 Hz, 3H); ^13^C NMR (151 MHz, CDCl_3_) δ 170.6, 170.0, 169.7, 169.1, 157.5, 151.5, 140.0, 135.6, 115.6, 114.2, 112.1, 109.8, 98.6, 77.7, 74.7, 73.1, 71.5, 62.9, 60.4, 21.1, 20.9, 20.7, 20.7; HRMS (ESI): mass calcd for C_23_H_28_O_11_[M + NH_4_]^+^, 498.1970; found, 498.1985.

#### 2.3.19. Synthesis of (E)-1″-(3-Acetoxy-5-O-2‴,3‴,6‴-Triacetyl-4‴-O-Methyl-β-D Glucopyranosidophenyl)- 2″-(4′-Acetoxyphenyl) Ethene (**24**)

To a solution of 4-iodophenylacetate **11** (147 mg, 0.56 mmol), compound **23** (270 mg, 0.56 mmol) in acetonitrile was added with palladium(II) acetate (0.006 mg, 0.028 mmol), benzyltriethylammonium chloride (128 mg, 0.56 mmol), and tributylamine (0.36 mL, 1.5 mmol) and stirred at 100 °C for 2 h under nitrogen. After 2 h, the mixture was cooled to room temperature, filtered through a short Celite pad, and then evaporated to dryness. The residue was taken up in dichloromethane, washed with diluted hydrochloric acid, water, and brine, dried over anhydrous MgSO_4_, filtered, and concentrated under reduced pressure. The residue was purified by silica gel column chromatography (ethyl acetate/n-hexane = 1:2) to yield product **24** (275 mg, 80%) as a white crystal. ^1^H NMR (600 MHz, CDCl_3_) δ 7.5 (d, *J* = 8.0 Hz, 2H), 7.1–7.0 (m, 3H), 7.0–6.9 (m, 3H), 6.6 (s, 1H), 5.3 (t, *J* = 9.2 Hz, 1H), 5.2 (t, *J* = 8.5 Hz, 1H), 5.1 (d, *J* = 7.6 Hz, 1H), 4.4 (d, *J* = 11.9 Hz, 1H), 4.3 (dd, *J* = 11.4, 5.8 Hz, 1H), 3.7 (s, 1H), 3.5 (s, 4H), 2.3 (s, 6H), 2.1 (s, 3H), 2.1 (s, 6H); ^13^C NMR (151 MHz, CDCl_3_) δ 170.6, 170.1, 169.7, 169.0, 156.8, 151.3, 139.7, 131.9, 129.8, 127.9, 126.0, 117.1, 116.8, 114.2, 98.7, 77.6, 74.8, 73.1, 71.6, 62.7, 60.5, 21.1, 20.9, 20.8, 20.7; HRMS (ESI): mass calcd for C_31_H_34_O_13_[M + H]^+^, 637.1999; found, 637.2014.

#### 2.3.20. Synthesis of 3-O-β-(4‴-O-Methylglucopyranosyl) Resveratrol (**3**)

Compound **24** (260 mg, 0.64 mmol) was dissolved in methanol (20 mL), and 0.2 M methanolic solution of sodium methoxide (20 mL) was added at room temperature. The resulting mixture was stirred for 1 h. The mixture was concentrated to dryness under reduced pressure. The residue was purified by silica gel column chromatography (methanol/dichloromethane = 1:8) to yield product **3** (217 mg, 84%) as a white powder. ^1^H NMR (600 MHz, (CD_3_)_2_CO) δ 7.36 (d, *J* = 7.9 Hz, 2H), 7.03 (d, *J* = 16.3 Hz, 1H), 6.85 (d, *J* = 16.3 Hz, 1H), 6.79 (d, *J* = 8.1 Hz, 2H), 6.72 (s, 1H), 6.64 (s, 1H), 6.41 (s, 1H), 4.86 (d, *J* = 7.7 Hz, 1H), 3.81 (d, *J* = 12.0 Hz, 1H), 3.65 (dd, *J* = 12.0, 4.6 Hz, 1H), 3.59 (t, *J* = 9.0 Hz, 1H), 3.52 (s, 3H), 3.46–3.37 (m, 2H), 3.16 (t, *J* = 9.3 Hz, 1H); ^13^C NMR (151 MHz, (CD_3_)_2_CO) δ 159.3, 158.8, 157.7, 139.9, 128.7, 127.9, 125.4, 115.6, 107.3, 105.3, 102.9, 100.8, 79.2, 77.1, 76.1, 74.1, 61.2, 59.6; HRMS (ESI): mass calcd for C_21_H_24_O_8_[M + NH_4_]^+^, 405.1544; found, 405.1551. The spectra of the above mentioned compounds is displayed in the part of Supplemental Material.

## 3. Results and Discussion

### 3.1. Retrosynthesis

Metabolite **2** and its regiomer **3** consist of a glycone attached to the aglycone moiety. The glycone moiety is 4-*O*-methyl glucopyranose, whereas the aglycone is a functional resveratrol featuring a stilbene core with a polyhydroxy group. Metabolite **2** is a structure in which 4-*O*-methylglucopyranose is attached to the 7-position hydroxyl group of resveratrol, whereas its regiomer **3** consists of a glycosyl moiety attached to the 3-position hydroxyl group of resveratrol. The synthesis of both metabolites involves a glycosylation reaction that introduces methylated glucose as the core reaction and the Heck reaction to form a stilbene skeleton [34].

**Scheme 1:**
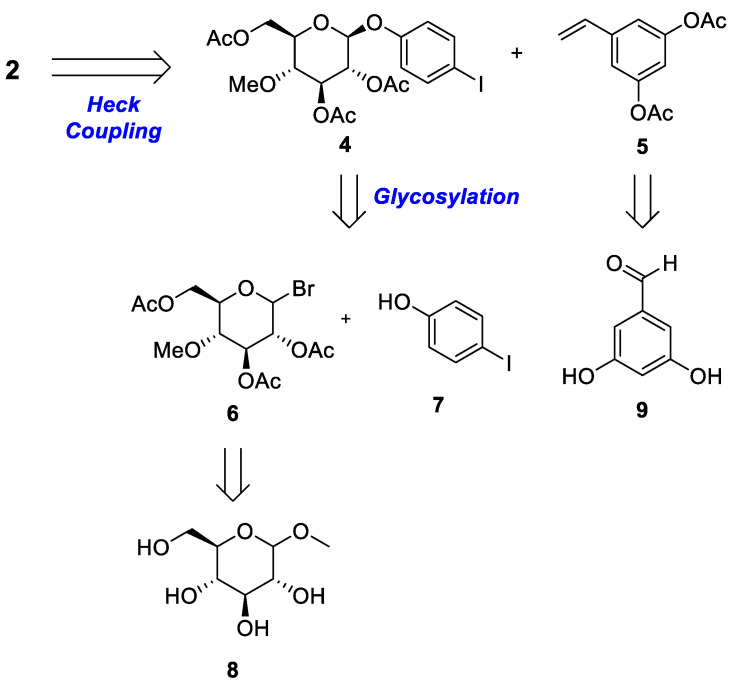
Retrosynthetic analysis of 4′-*O*-*β*- (4‴-*O*-methylglucopyranosyl)resveratrol **2**.

**Scheme 2:**
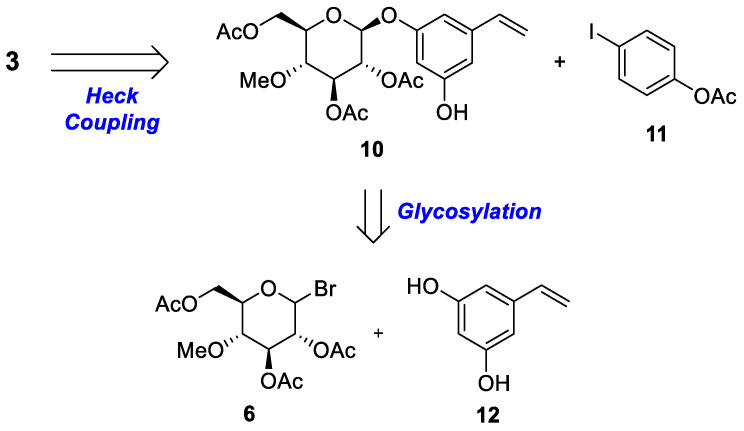
Retrosynthetic analysis of 3-*O*-*β*-(4‴-*O*-methylglucopyranosyl)resveratrol **3**.

Scheme 1 and Scheme 2 provide a retrosynthetic methodology for the synthesis of both metabolites **2** and **3**. Stilbene moiety **2** and its regiomer **3** were constructed via palladium-catalyzed Heck coupling. The rate-limiting step of the glycosylation reaction was performed with selectively protected compound **6** and commercially available iodophenol **7**. Compound **10** was obtained from the glycosylation of compound **6** and styrene **12**, which was synthesized from readily available dihydroxy benzaldehyde **9**.

### 3.2. Chemistry

**Scheme 3:**
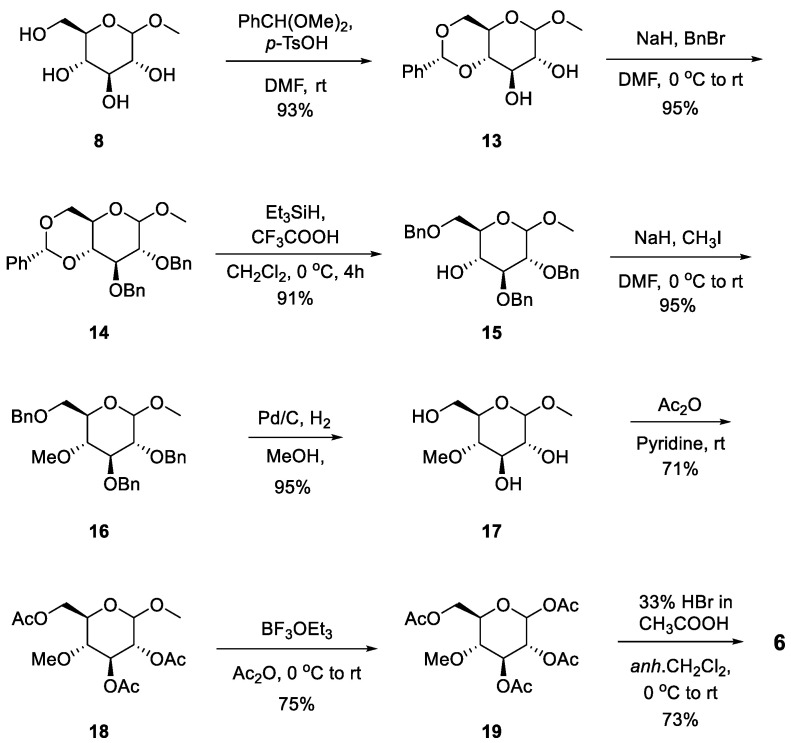
Synthesis of 4-O-methylglucopyransyl bromide **6**.

The reaction commenced with the preparation of the glycosyl donor, 4-O-methylglycopyranosyl bromide **6**, as shown in Scheme 3, which involves eight steps from commercially available methyl-α-D-glucopyranoside **8**. Regioselective protection of 4, 6-diol from the starting material was accomplished by the introduction of a 4,6-O-benzylidene group using benzaldehyde dimethyl acetal under acidic conditions, yielding protected compound **13**. 2,3-Di-O-benzylation of **13** generated **14** using NaH and BnBr. Further regioselective opening of the benzylidene ring of intermediate **14** was conducted with the help of triethylsilane (TES) and trifluoroacetic acid (TFA) to obtain alcohol **15** [35]. Methylation of compound **15** with NaH and MeI in N, N-dimethylformamide yielded product **16**, followed by hydrogenolysis to yield product **17**. Acetylation of the hydroxy groups of **17** was performed using pyridine and acetic anhydride to yield **18**, followed by the replacement of an anomeric methoxy group with an acetoxy group using boron trifluoride diethyl etherate to yield **19**. Finally, grafting of the anomeric acetoxy group was performed to incorporate bromine using HBr (33% in acetic acid) to yield acylated glycosyl bromide **6** at 73% [36]. As the final compound, 4-O-methylglucopyranosyl bromide **6**, has poor chemical stability, it is suitable to obtain a large amount of acetate compound **19** and synthesize **6** immediately when necessary.

**Scheme 4:**
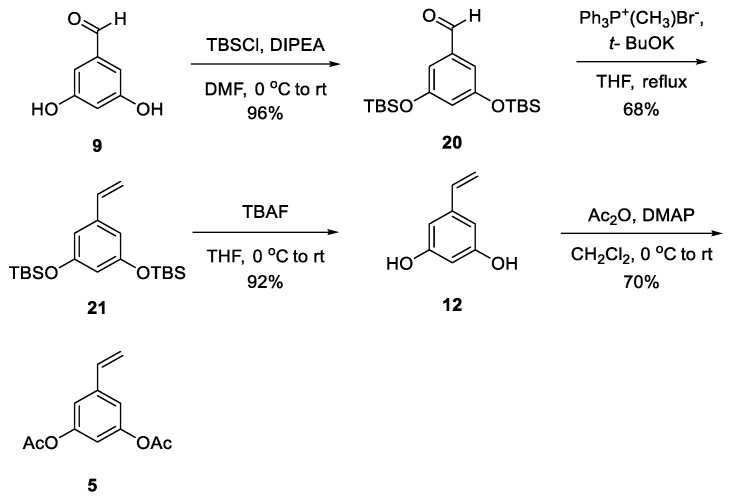
Synthesis of diacetoxystyrene **5** and dihydroxystyrene **12**.

3,5-Dihydroxystyrene **12** and 3,5 diacetoxyystyrene **5** were synthesized from commercially available 3,5-dihydroxy benzaldehyde **9** according to Scheme 4. Protection of the hydroxy group of **9** with TBDMS yielded **20**, and the Wittig reaction yielded olefin **21** using methyltriphenylphosphonium bromide under basic conditions. The TBDMS group in intermediate **21** was removed using tetrabutylammonium fluoride (TBAF) to furnish dihydroxy styrene **12**, and further acetylation of both hydroxy groups resulted in **12** at 70% yield.

After obtaining **6** and **12**, the next target was to synthesize substrates **4** and **10**, which participate in the Heck reaction for the synthesis of the stilbene core.

We performed glycosylation of both iodophenol **7** and dihydroxystyrene **12** separately with 4-*O*-methylglycopyranosyl bromide **6** under different reaction conditions, as shown in Table 1 and Table 2. Using Ag_2_CO_3_ in acetonitrile produced low-yield glycoside products **4** and **10** up to 16% and 19%, respectively. We attempted to improve glycosylation using a phase transfer catalyst (TBAB) in a two-phase system (aqueous NaOH and K_2_CO_3_) in CHCl_3_. Unfortunately, the reaction yielded trace amounts. The reaction was incomplete, and the substrate was recovered for reuse. Bromide compound **6** can be decomposed into glycal by an alkaline water phase and phenoxide anion [37]. Therefore, excess use of water in the reaction lowers the yield of the compound. After utilizing several conditions (Table 1 and Table 2), the glycosylation reaction under the phase transfer catalyst BnNBu_3_Cl and K_2_CO_3_ as a base at room temperature yielded product **4** at 57% yield. The desired mono-glucosylated product **10** was obtained at 40% yield along with the undesired di-glucosylated product as a mixture, which was separated by column chromatography.

Aryl iodide **7** was the choice for palladium-catalyzed Heck coupling because of its greater reactivity over other aryl halides.

The palladium-catalyzed Heck reaction between glycosylated compounds **4** and **5** under (Pd(OAc)_2_, BnEt_3_N^+^Cl^−^, Bu_3_N) in warm acetonitrile produced **22** at 55% yield. Deprotection of the acetyl protecting groups of **22** under a methanolic solution of sodium methoxide resulted in metabolite **2** at 85% yield.

**Scheme 5:**
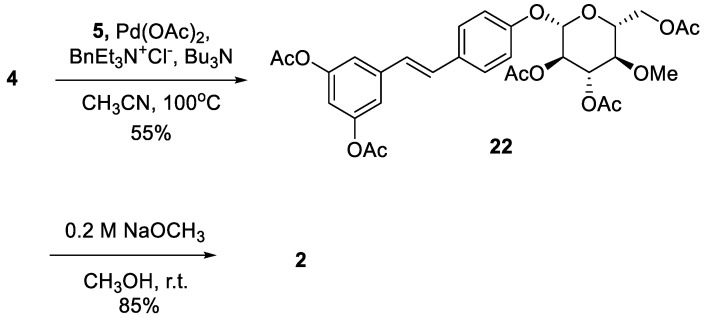
Synthesis of 4′-*O*-*β*-(4‴-*O*-methylglucopyranosyl)resveratrol (**2**).

**Scheme 6:**
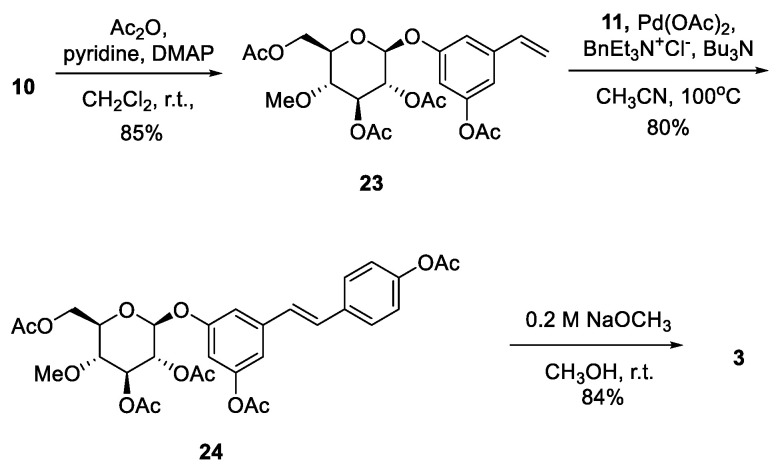
Synthesis of 3-*O*-*β*-(4‴-*O*-methylglucopyranosyl)resveratrol (**3**).

The hydroxy group of glycosylated product **10** was protected by acetylation to yield **23**. Similar to Scheme 5, the protected product **23** undergoes a palladium-catalyzed Heck reaction with 4-iodophenyl acetate **11** to build styrene compound **24** at 80% yield. Finally, basic hydrolysis of the acetyl protecting groups under a methanolic solution of sodium methoxide afforded another metabolite, **3**, at 84% yield (Scheme 6).

## 4. Conclusions

In conclusion, an efficient total synthesis was performed for the preparation of resvebassianol A (**2**, a metabolite of resveratrol by *Beauveria bassiana*) and its regiomer (**3**) through glycosylation and a palladium-catalyzed Heck reaction. Resvebassianol A and regiomer **3** were synthesized in 11 and 12 linear steps, with overall yields of 7.5% and 6.3%, respectively. Incorporation of 4-*O* methyl glyosyl was performed through the glycosylation reaction and was optimized using a phase transfer catalyst with varying bases. This resulted in an elevated yield of up to 40% and 57%, respectively. Thus, this method can be helpful for the synthesis of metabolites that are difficult to obtain from plant sources and through microbial biotransformation. This strategy can also be used for the synthesis of other related metabolites.

## Figures and Tables

**Table 1:**
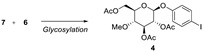
Optimization of glycosylation reaction for synthesis of **4 ^a^**.

Entry ^a^	Base	Reagent	Solvent	Temp. (°C)	Yield (%) ^d^
1		Ag_2_CO_3_ (1eq)	CH_3_CN	r.t	16
2 ^b^	NaOH	TBAB	CHCl_3_:H_2_O (1:1)	45	14
3 ^b^	K_2_CO_3_	TBAB	CHCl_3_:H_2_O (1:1)	45	17
4 ^c^	K_2_CO_3_	BnNBu_3_Cl	CHCl_3_	r.t	57

**^a^** Reaction was carried out using 1 equiv of both starting materials **7** and **6**. ^b^ Base (1.1 eq), TBAB (0.2 eq), ^c^ Base (2.5 eq), and BnNBu_3_Cl (0.1 eq). ^d^ Isolated yield.

**Table 2:**
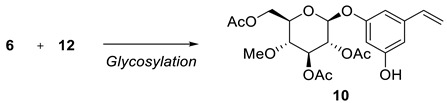
Optimization of glycosylation reaction for synthesis of **10 ^a^**.

Entry	Base	Reagent	Solvent	Temp. (°C)	Yield (%) ^d^
1		Ag_2_CO_3_	CH_3_CN	r.t	19
2 ^b^	NaOH	TBAB	CHCl_3_:H_2_O (1:1)	45	13
3 ^b^	K_2_CO_3_	TBAB	CHCl_3_:H_2_O (1:1)	45	15
4 ^c^	K_2_CO_3_	BnNBu_3_Cl	CHCl_3_	r.t	40

**^a^** Reaction was carried out using 1 equiv of both starting materials **6** and **12**. ^b^ Base (1.1 eq), TBAB (0.2 eq), ^c^ Base (2.5 eq), and BnNBu_3_Cl (0.1 eq). ^d^ Isolated yield.

## Data Availability

Data is contained within the article or supplementary material.

## Supplementary Materials

The following are available online at https://www.mdpi.com/article/10.3390/antiox10101509/s1, Figure S1: ^1^H-NMR (600 MHz, CDCl_3_) of compound **13**, Figure S2: ^13^C-NMR (150 MHz, CDCl_3_) of compound **13**, Figure S3: ^1^H-NMR (600 MHz, CDCl_3_) of compound **14**, Figure S4: ^13^C-NMR (150 MHz, CDCl_3_) of compound **14**, Figure S5: ^1^H-NMR (600 MHz, CDCl_3_) of compound **15**, Figure S6: ^1^C-NMR (150 MHz, CDCl_3_) of compound **15**, Figure S7: ^1^H-NMR (600 MHz, CDCl_3_) of compound **16**, Figure S8: ^13^C-NMR (150 MHz, CDCl_3_) of compound **16**, Figure S9: ^1^H-NMR (600 MHz, CD_3_OD-d4) of compound **17**, Figure S10: ^1^H-NMR (600 MHz, CD_3_OD-d4) of compound **17**, Figure S11: ^1^H-NMR (600 MHz, CDCl_3_) of compound **18**, Figure S12: ^13^C-NMR (150 MHz, CDCl_3_) of compound **18**, Figure S13: ^1^H-NMR (600 MHz, CDCl_3_) of compound **19**, Figure S14: ^13^C-NMR (150 MHz, CDCl_3_) of compound **19**, Figure S15: ^1^H-NMR (600 MHz, CDCl_3_) of compound **6**, Figure S16: ^13^C-NMR (150 MHz, CDCl_3_) of compound **6**, Figure S17: ^1^H-NMR (600 MHz, CDCl_3_) of compound **20**, Figure S18: ^13^C-NMR (150 MHz, CDCl_3_) of compound **20**, Figure S19: ^1^H-NMR (600 MHz, CDCl_3_) of compound **21**, Figure S20: ^13^C-NMR (150 MHz, CDCl_3_) of compound **21**, Figure S21: ^1^H-NMR (600 MHz, CD_3_OD-d4) of compound **12**, Figure S22: ^13^C-NMR (150 MHz, CD_3_OD-d4) of compound **12**, Figure S23: ^1^H-NMR (600 MHz, CDCl_3_) of compound **5**, Figure S24: ^13^C-NMR (150 MHz, CDCl_3_) of compound **5**, Figure S25: ^1^H-NMR (600 MHz, CDCl_3_) of compound **11**, Figure S26: ^13^C-NMR (150 MHz, CDCl_3_) of compound **11**, Figure S27: ^1^H-NMR (600 MHz, CDCl_3_) of compound **4**, Figure S28: ^13^C-NMR (150 MHz, CDCl_3_) of compound **4**, Figure S29: ^1^H-NMR (600 MHz, CDCl_3_) of compound **22**, Figure S30: ^13^C-NMR (150 MHz, CDCl_3_) of compound **22**, Figure S31: ^1^H-NMR (600 MHz, (CD3)2CO)) of compound **2**, Figure S32: ^13^C-NMR (150 MHz, (CD_3_)_2_CO) of compound **2**, Figure S33: ^1^H-NMR (600 MHz, CDCl_3_) of compound **10**, Figure S34: ^13^C-NMR (150 MHz, CDCl_3_) of compound **10**, Figure S35: ^1^H-NMR (600 MHz, CDCl_3_) of Compound **23**, Figure S36: ^13^C-NMR (151 MHz, CDCl_3_) of Compound **23**, Figure S37: ^1^H-NMR (600 MHz, CDCl_3_) of compound **24**, Figure S38: ^13^C-NMR (150 MHz, CDCl_3_) of compound **24**, Figure S39: ^1^H-NMR (600 MHz, (CD_3_)_2_CO) of compound **3**, Figure S40: ^13^C-NMR (150 MHz, (CD_3_)_2_CO) of compound **3**.
