# Supplementary material for: Total Synthesis of Resvebassianol A, a Metabolite of Resveratrol by Beauveria bassiana
Source: Antioxidants (Basel). 2021 Sep 23;10(10):1509. doi: 10.3390/antiox10101509 (PMC8532873; doi:10.3390/antiox10101509)
Supplement: Supplementary file 1 [file antioxidants-10-01509-s001.zip › antioxidants-1370518-supplementary.pdf]

Supporting Information

Total Synthesis of Resvebassianol A, a Metabolite of Resveratrol by *Beauveria bassiana*

Om Darlami and Dongyun Shin *

College of Pharmacy, Gachon Univeristy, 191. Hambakmoe-ro, Yeonsu-gu, Incheon 21936, Korea
*Correspondence: dyshin@gachon.ac.kr;

Contents:

^{1}H and ^{13}C-NMR spectra of the compounds,

[1] H-NMR (600MHz, $CDCl_3$) of compound 13

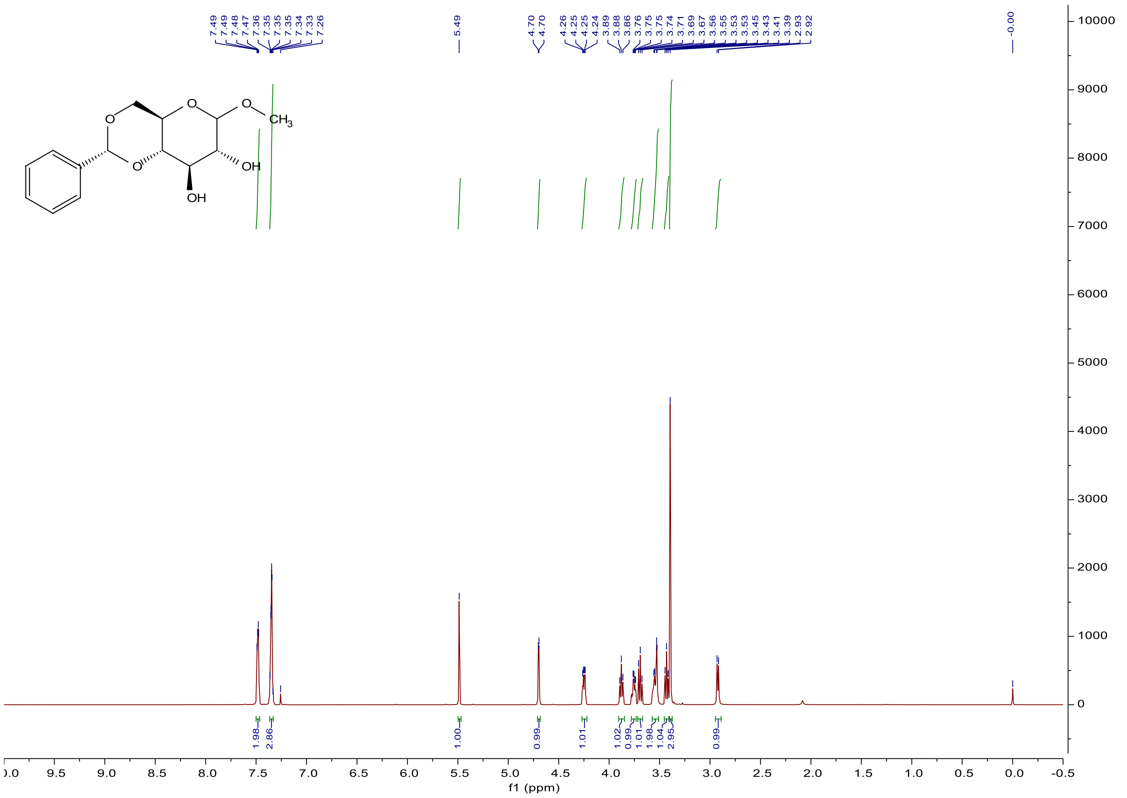

^{13}C-NMR (150MHz, $CDCl_3$) of compound 13

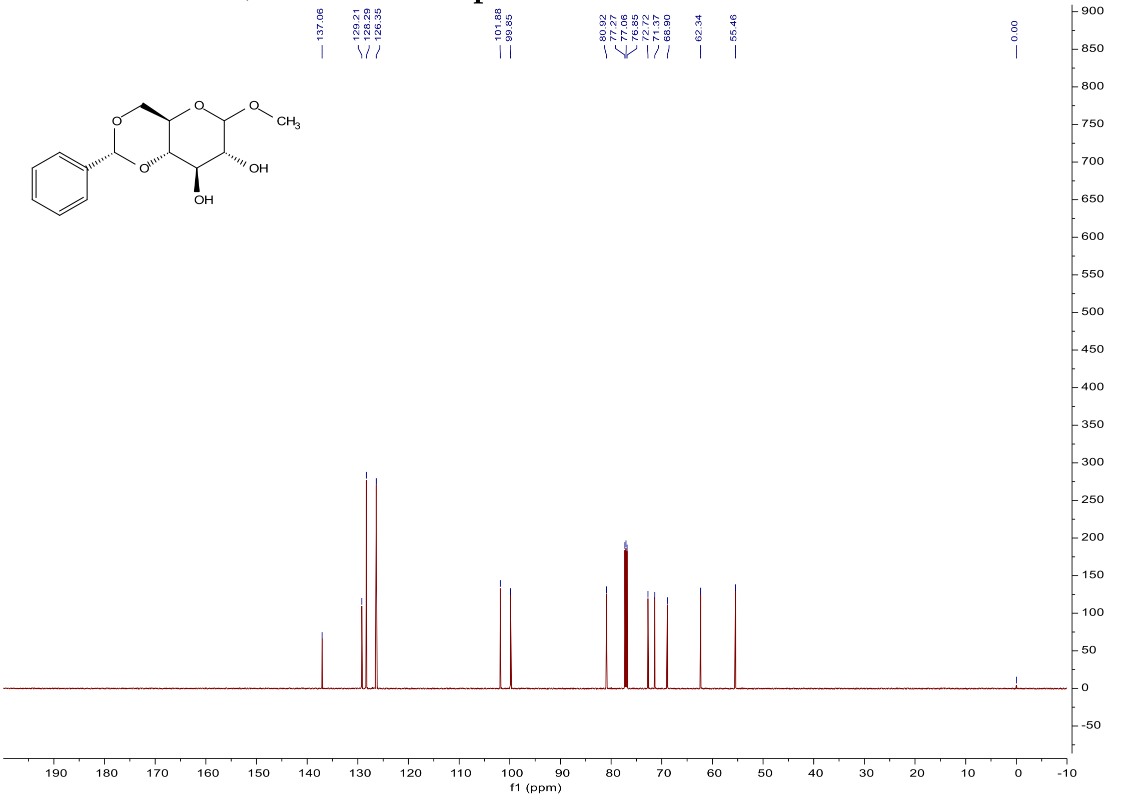

^{1}H-NMR (600MHz, $CDCl_3$) of compound 14

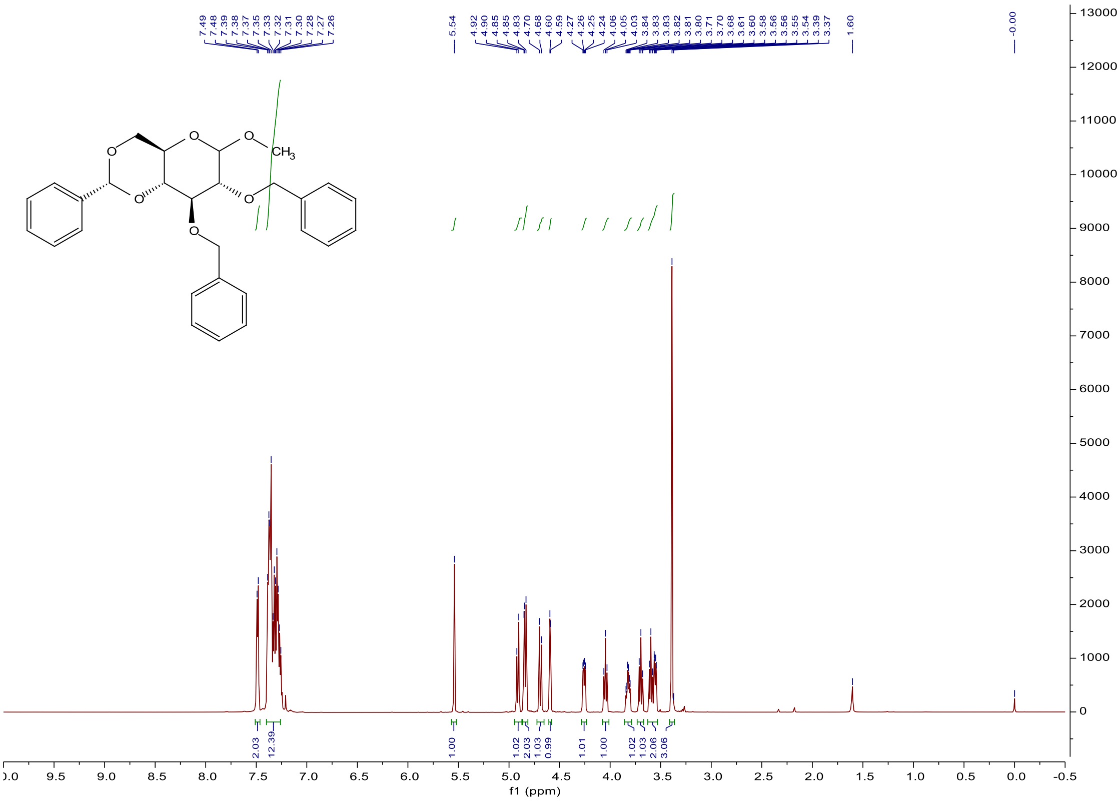

^{13}C-NMR (150 MHz, $CDCl_3$) of compound 14

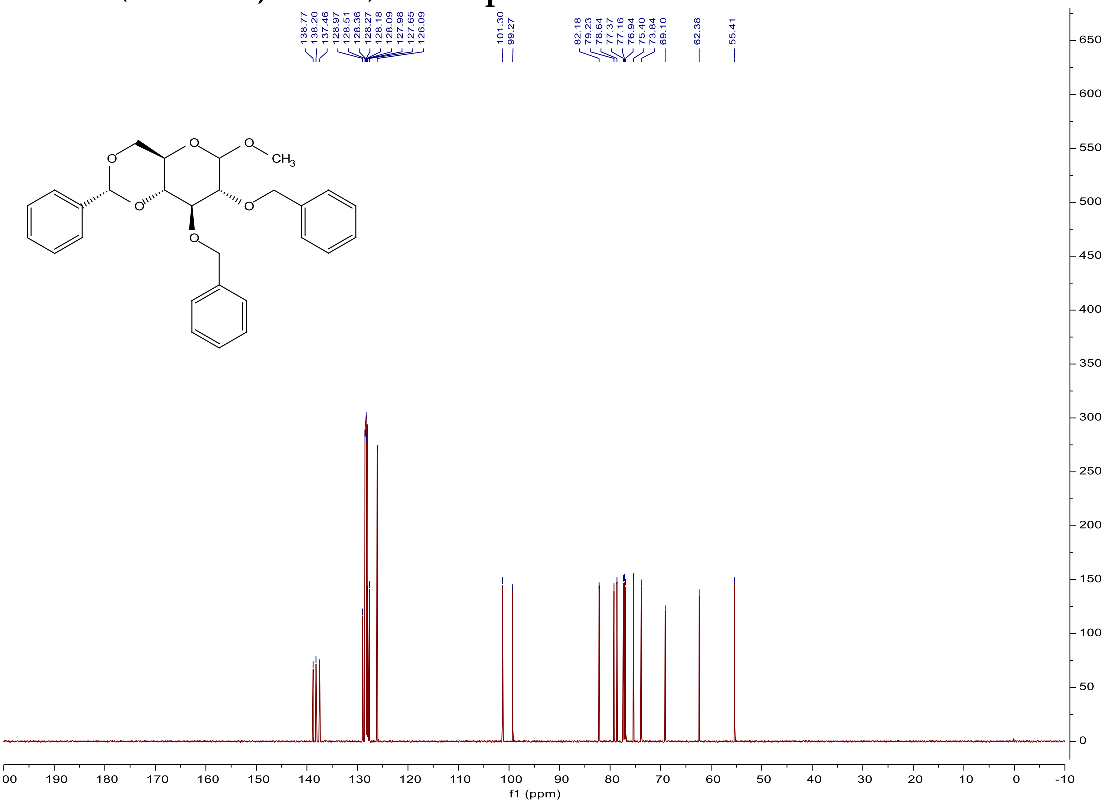

[1] H-NMR (600MHz, $CDCl_3$) of compound 15

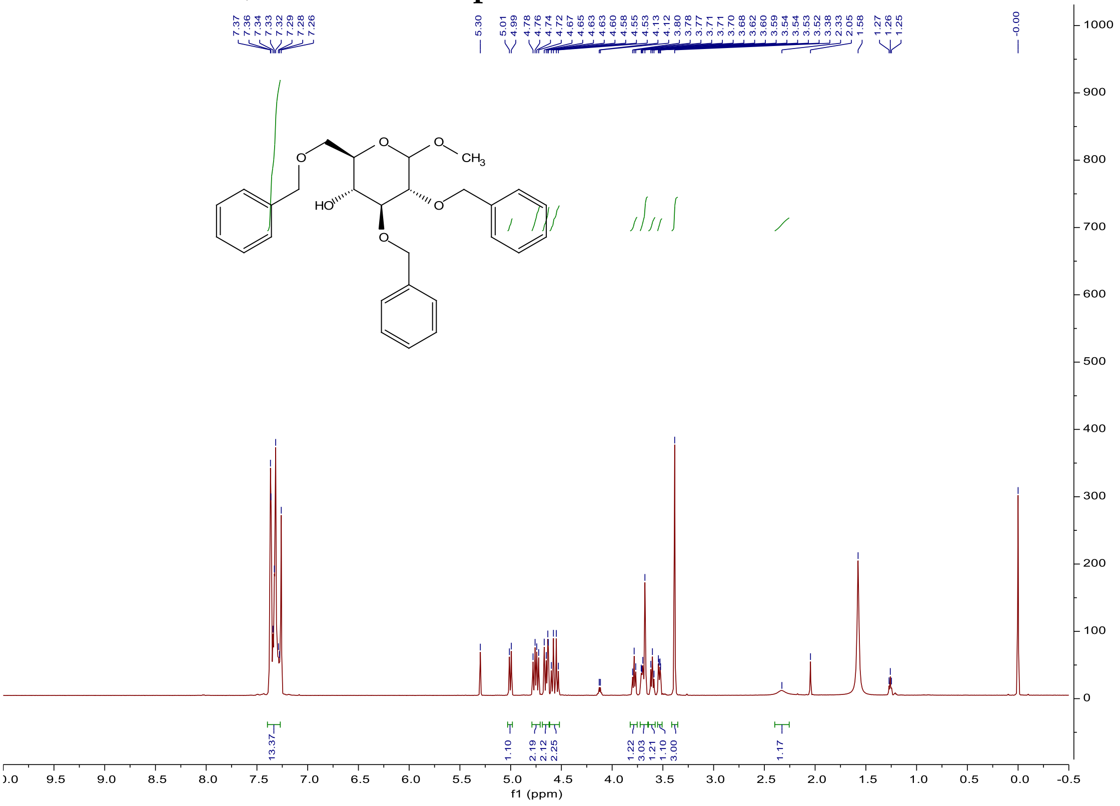

[1] C-NMR (150MHz, $CDCl_3$) of compound 15

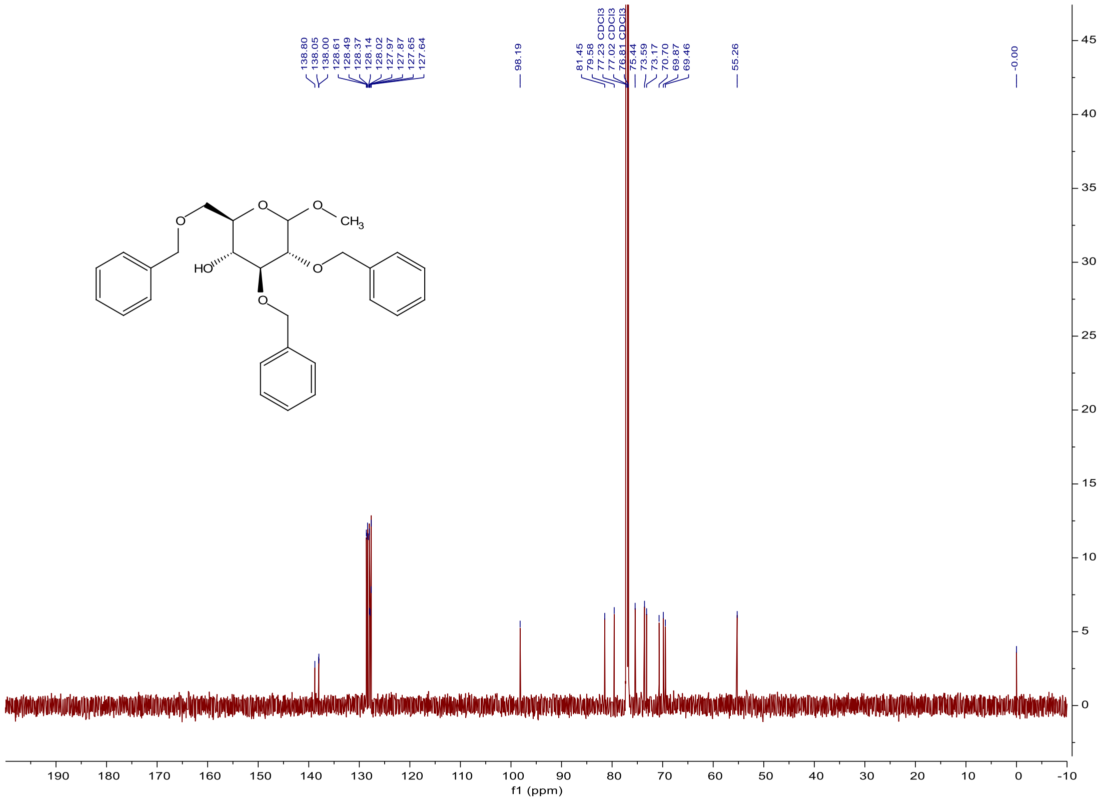

^{1}H-NMR (600MHz, $CDCl_3$) of compound 16

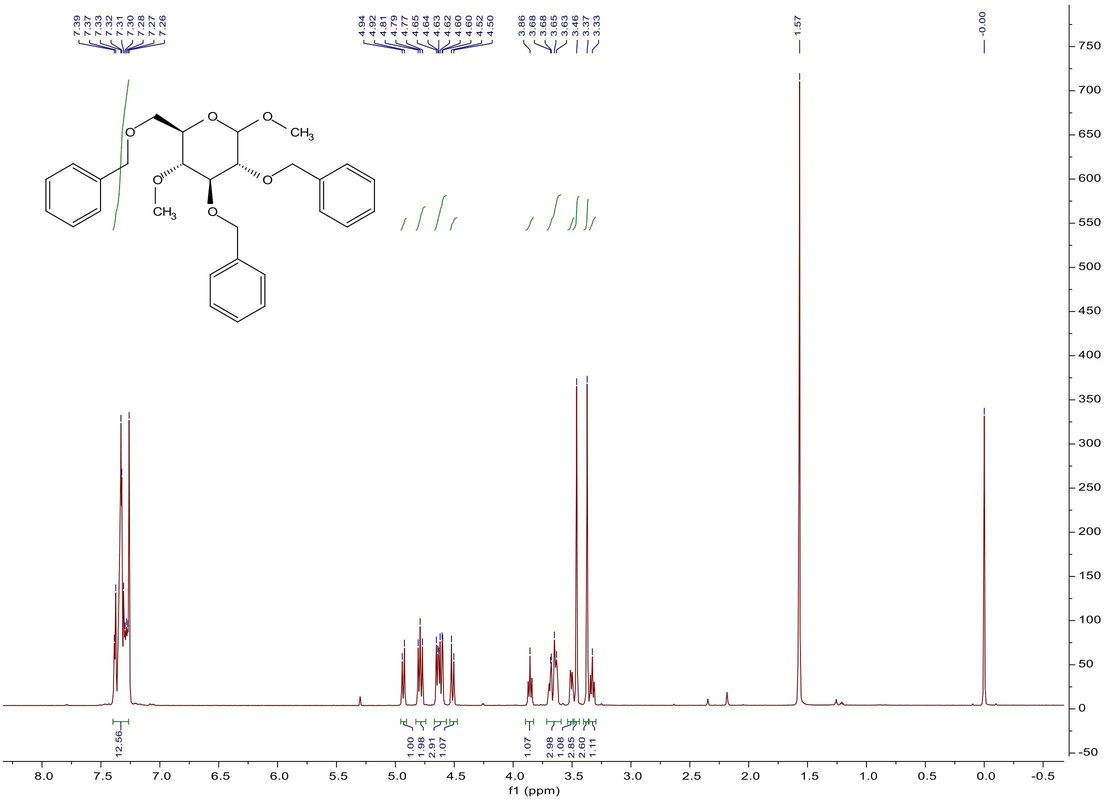

^{13}C-NMR (150MHz, $CDCl_3$) of compound 16

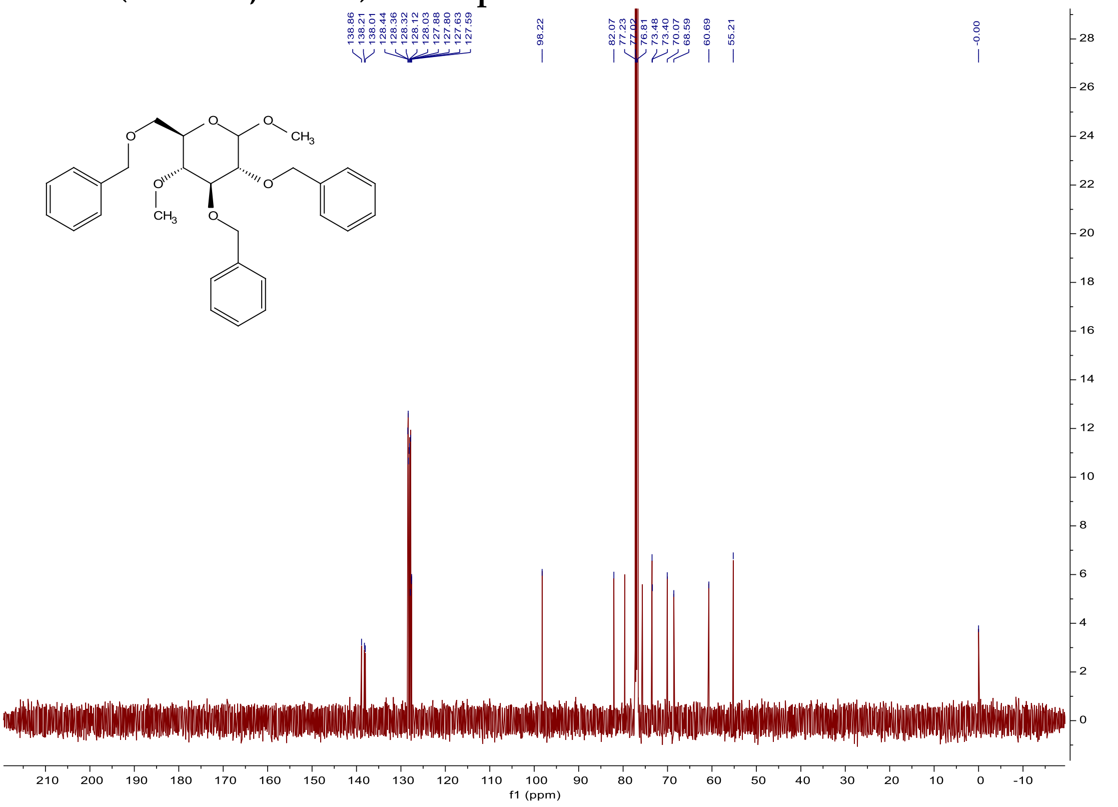

¹H-NMR (600 MHz, CD_3OD-*d*4) of compound 17

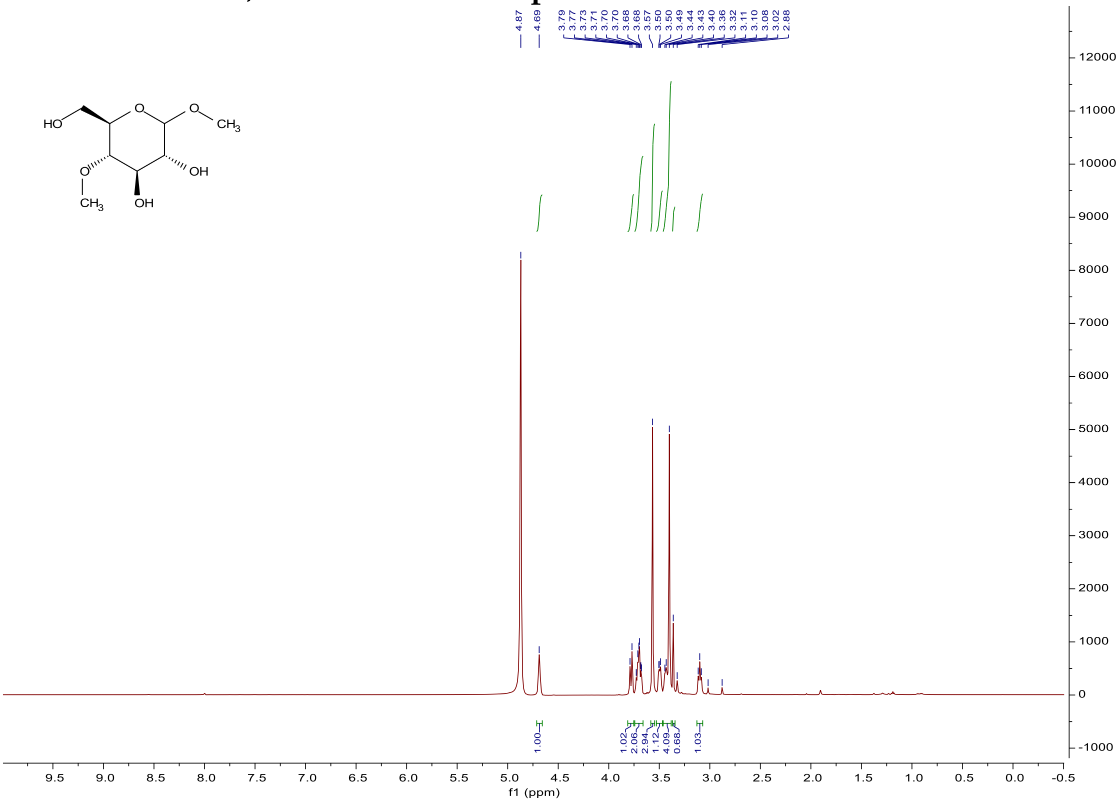

¹H-NMR (600 MHz, CD_3OD-*d*4) of compound 17

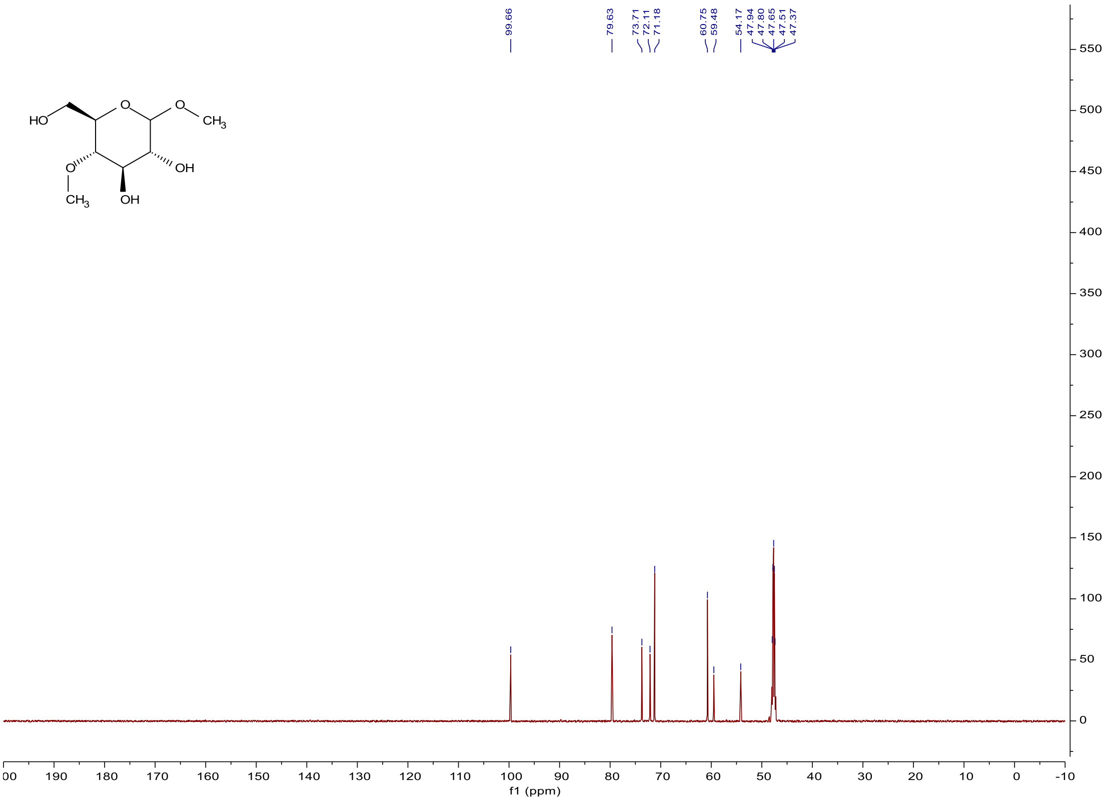

^{1}H-NMR (600MHz, $CDCl_3$) of compound 18

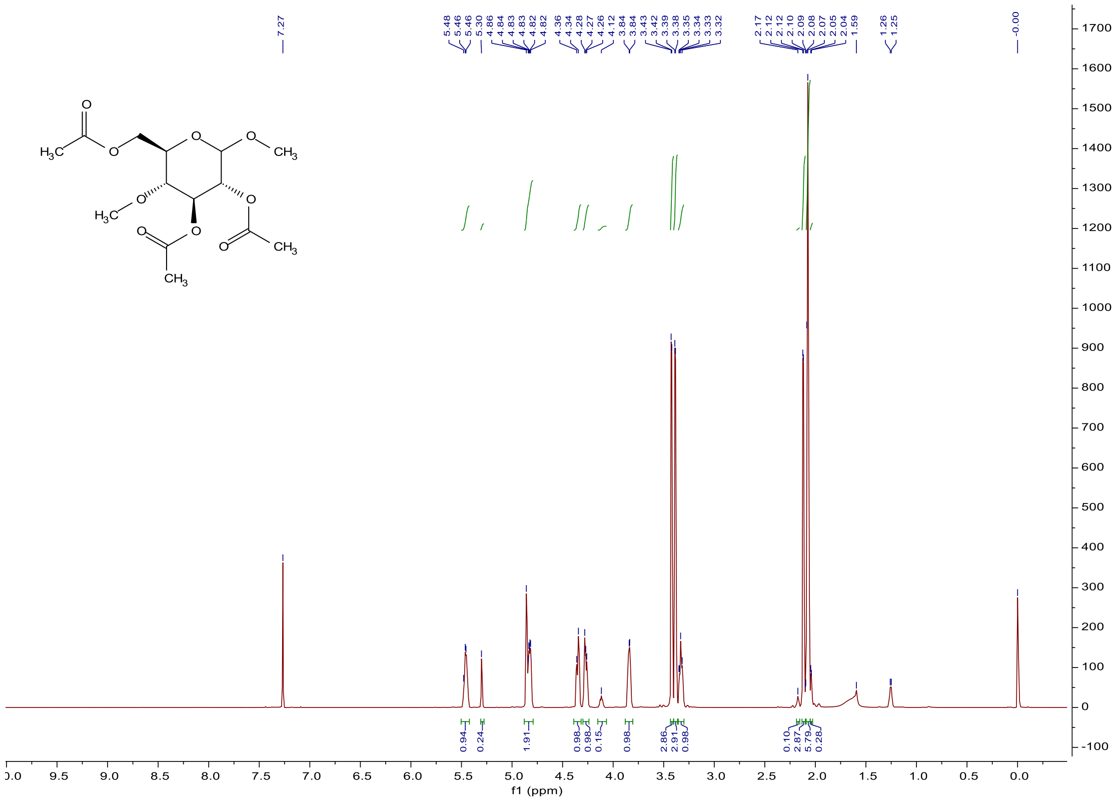

^{13}C-NMR (150MHz, $CDCl_3$) of compound 18

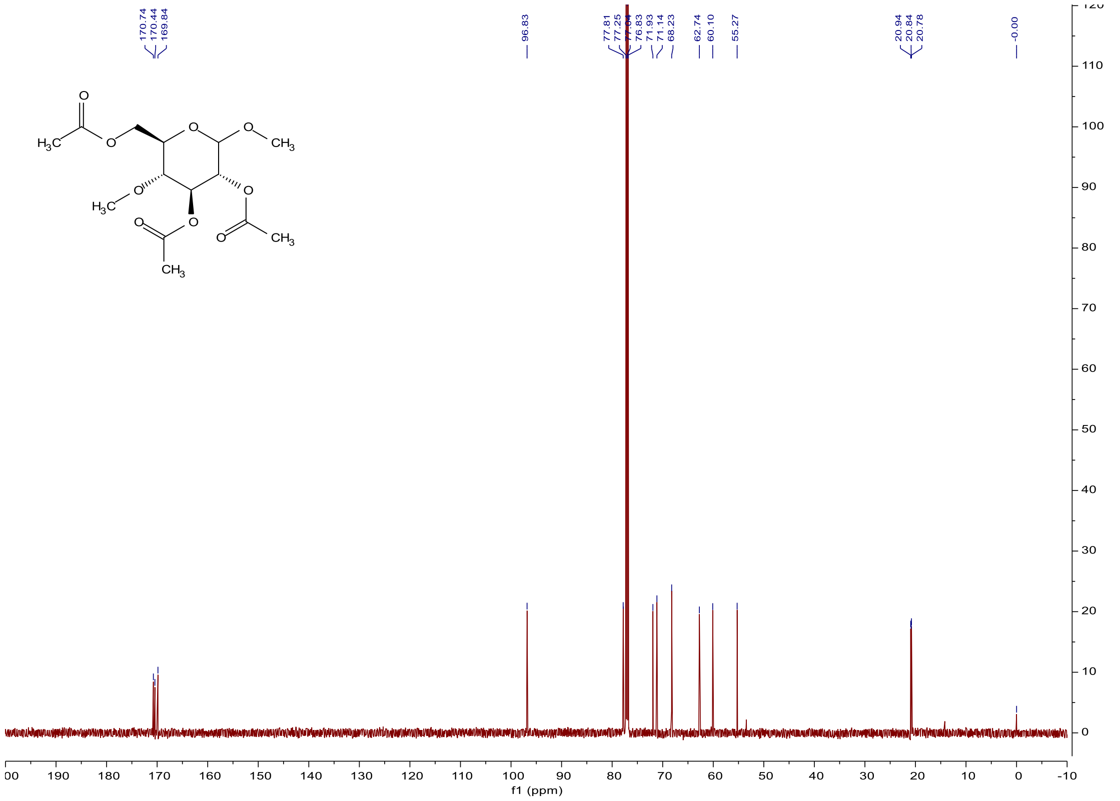

^{1}H-NMR (600MHz, $CDCl_3$) of compound 19

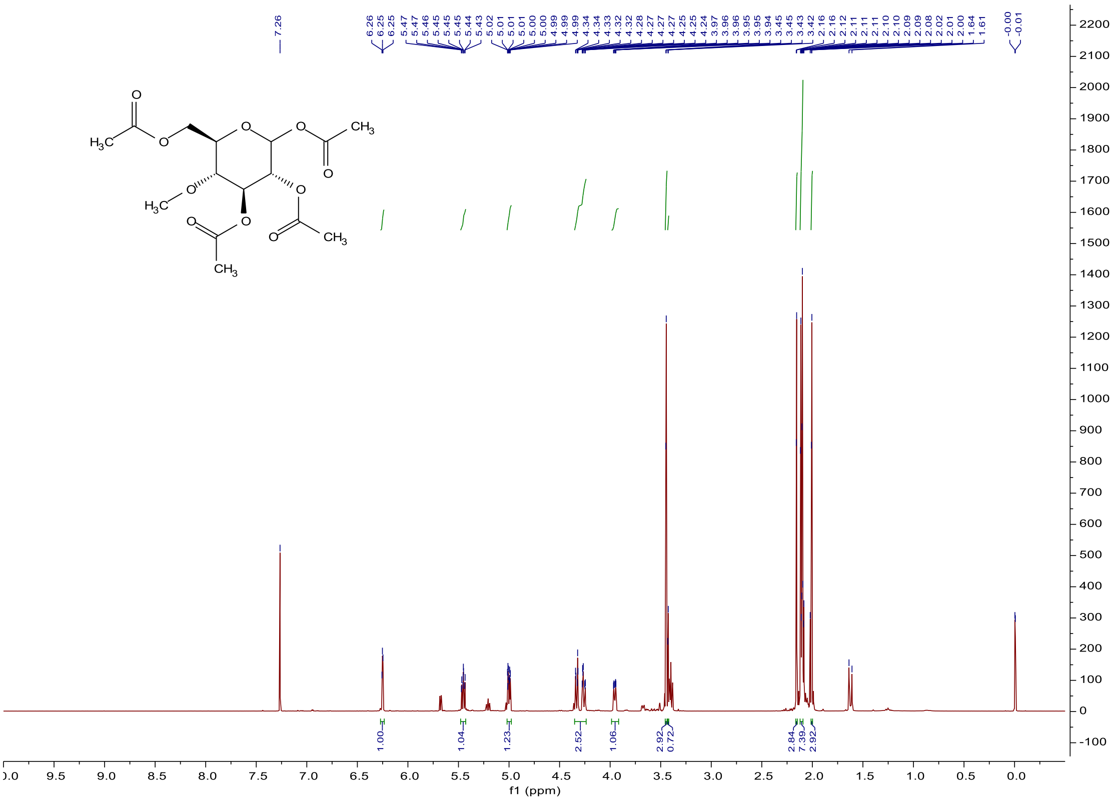

^{13}C-NMR (150MHz, $CDCl_3$) of compound 19

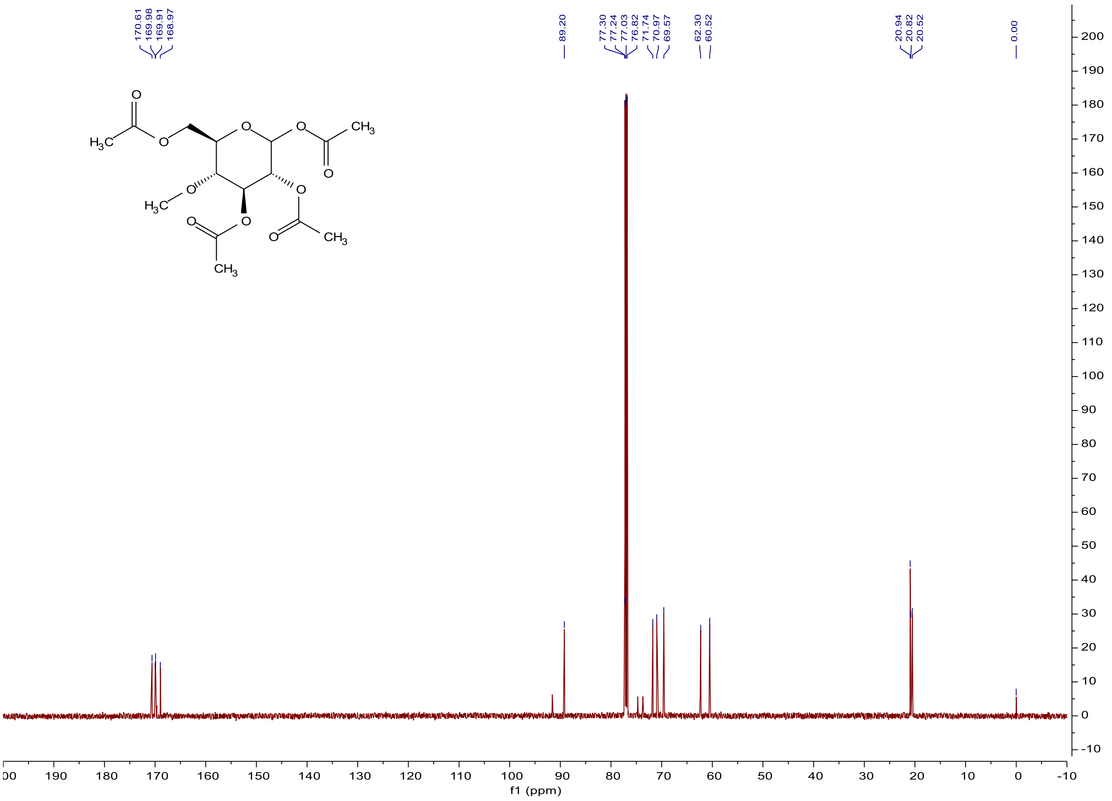

¹H-NMR (600MHz, CDCl₃) of compound 6

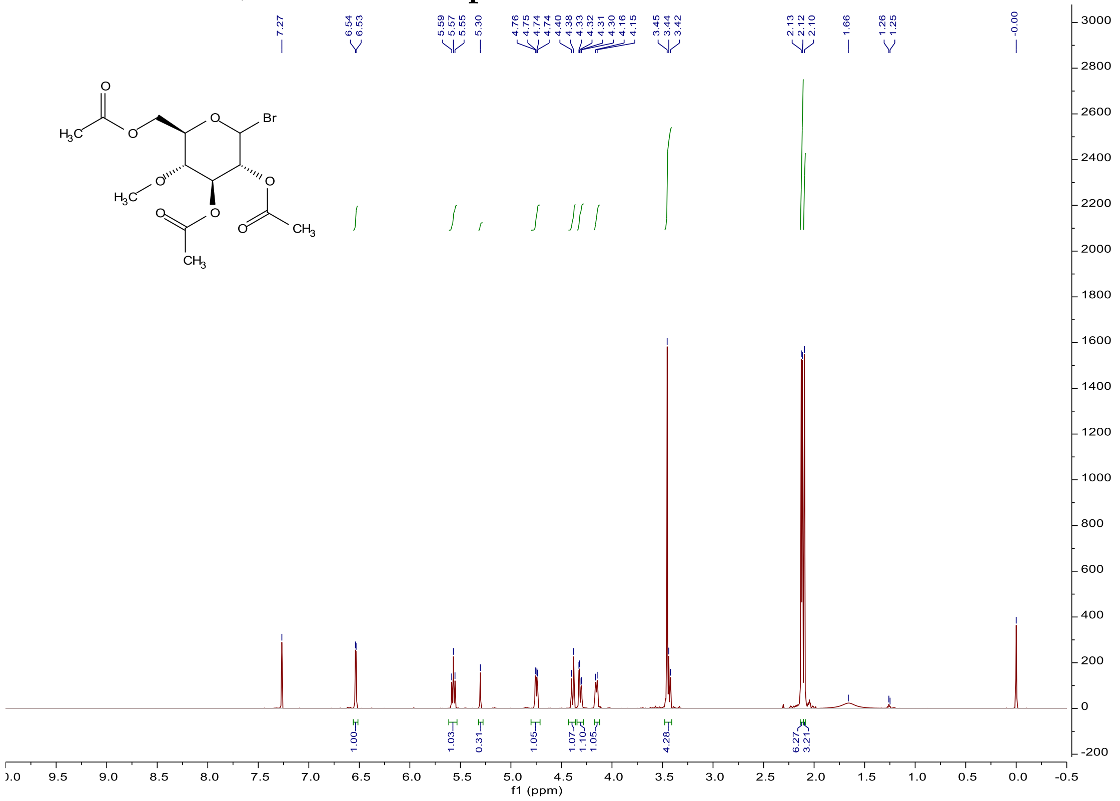

¹³C-NMR (150MHz, CDCl₃) of compound 6

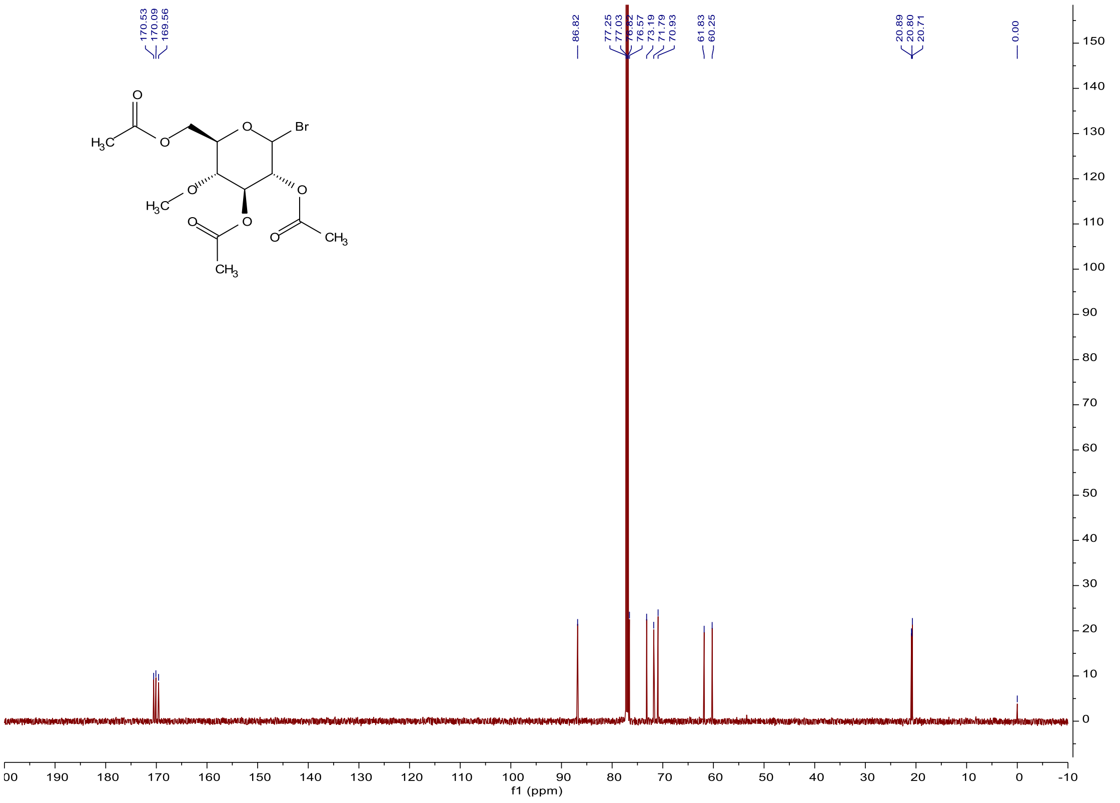

^{1}H-NMR (600MHz, $CDCl_3$) of compound 20

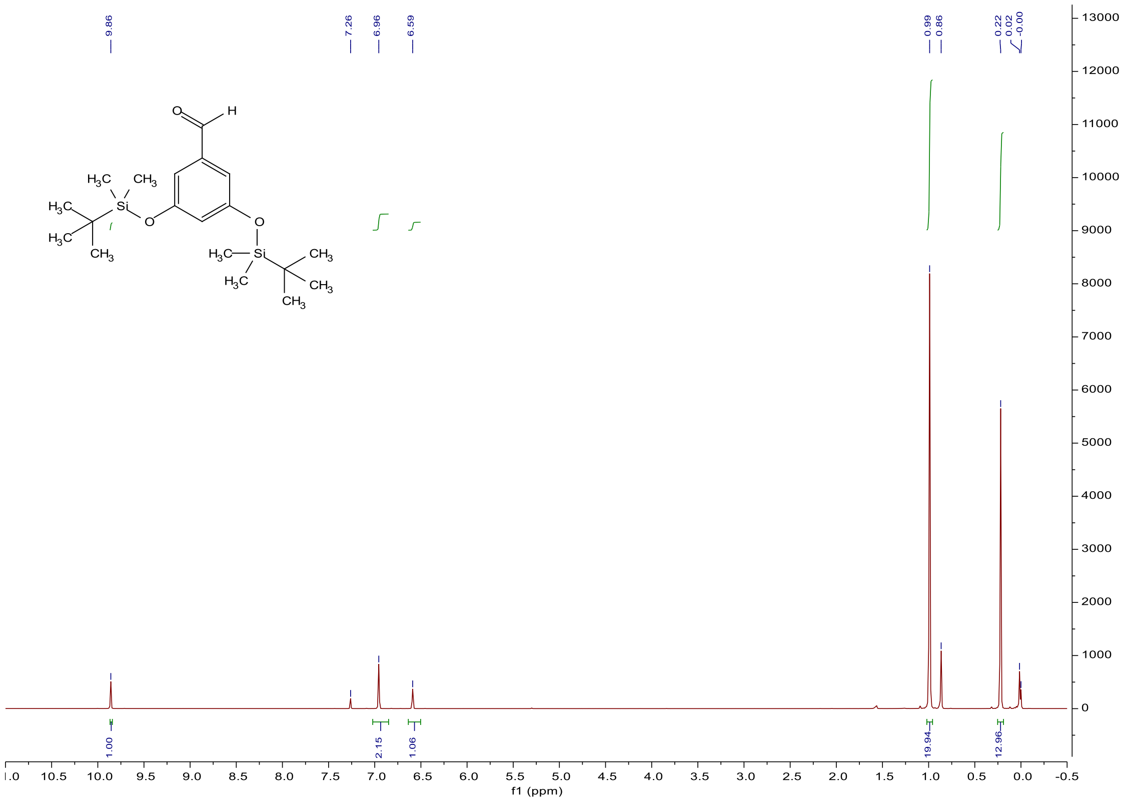

^{13}C-NMR (150 MHz, $CDCl_3$) of compound 20

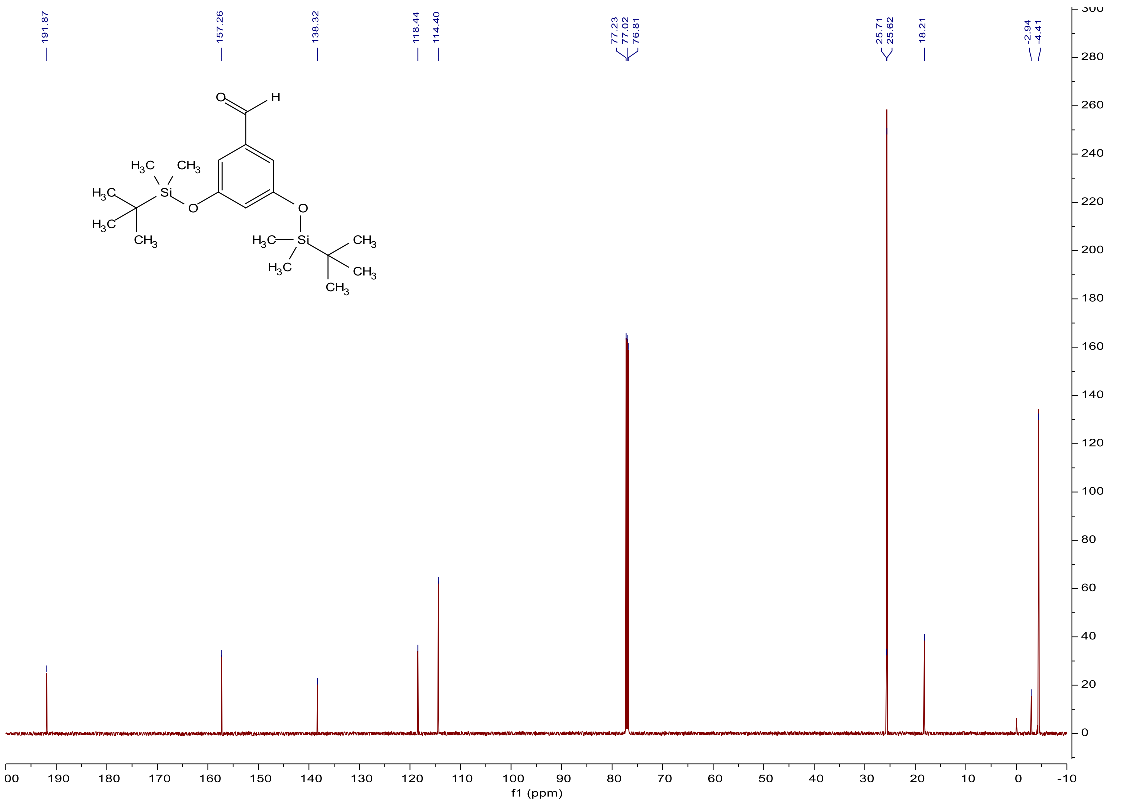

^{1}H-NMR (600MHz, $CDCl_3$) of compound 21

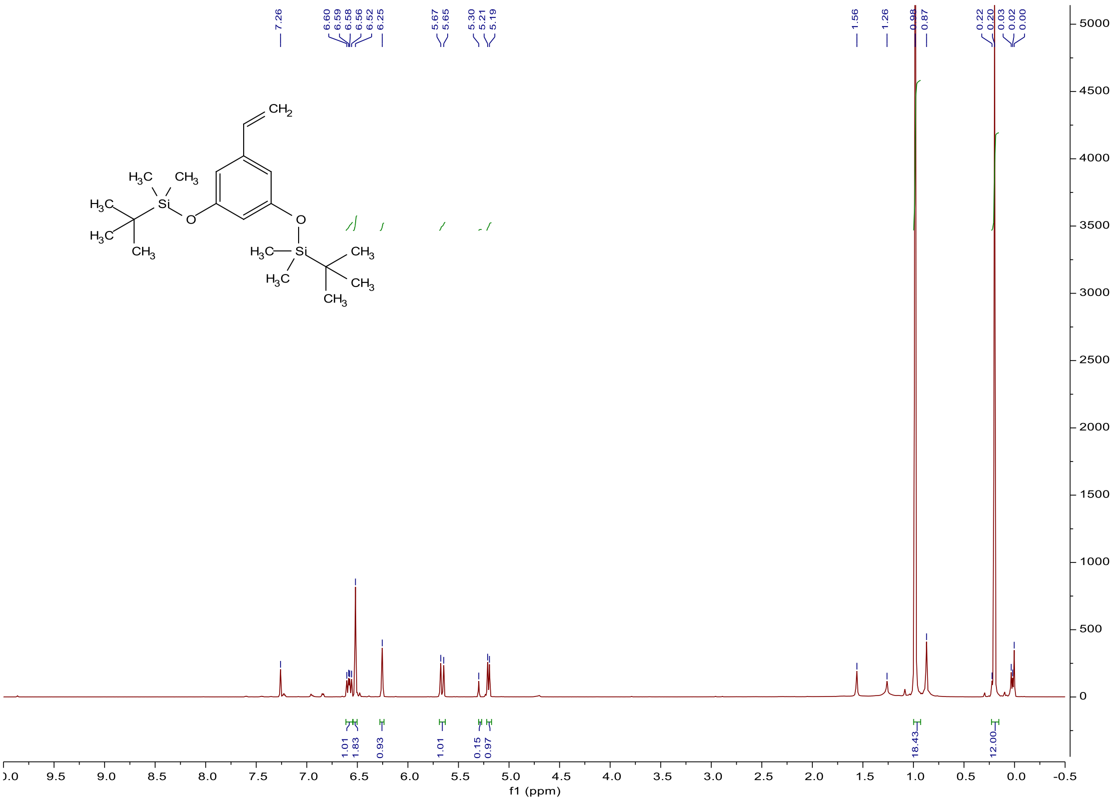

^{13}C-NMR (150MHz, $CDCl_3$) of compound 21

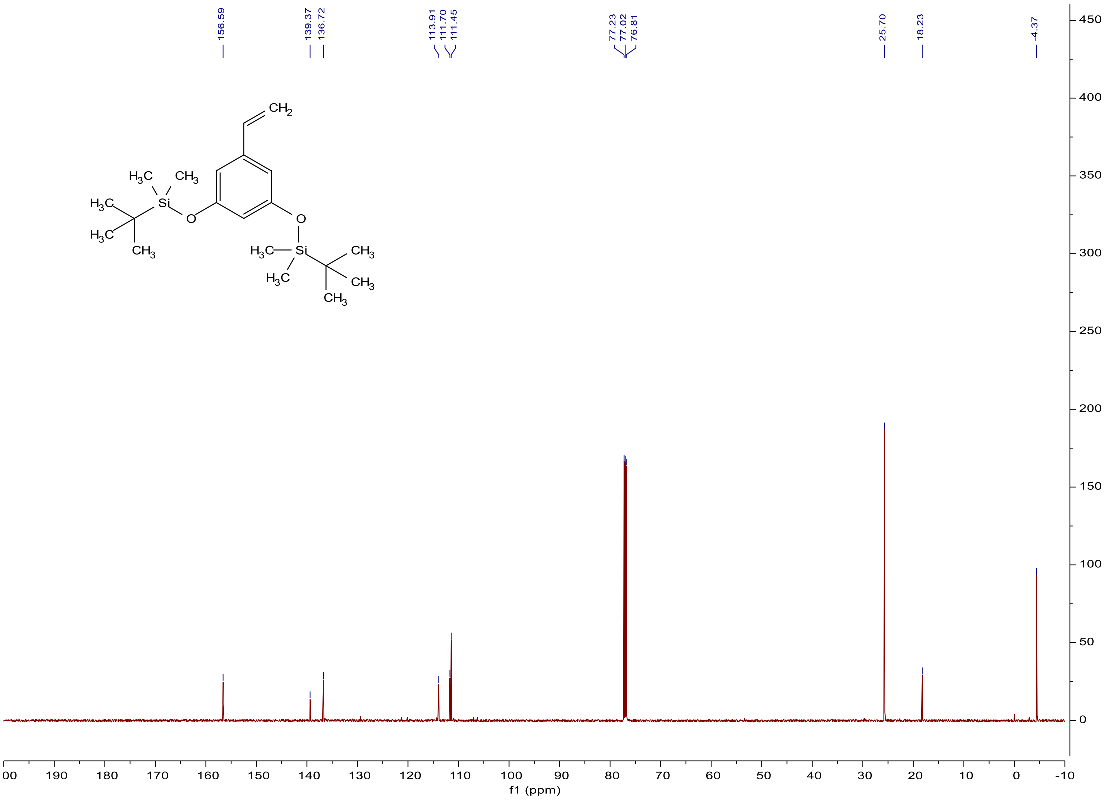

^{1}H-NMR (600MHz, CD_3OD-d_4) of compound 12

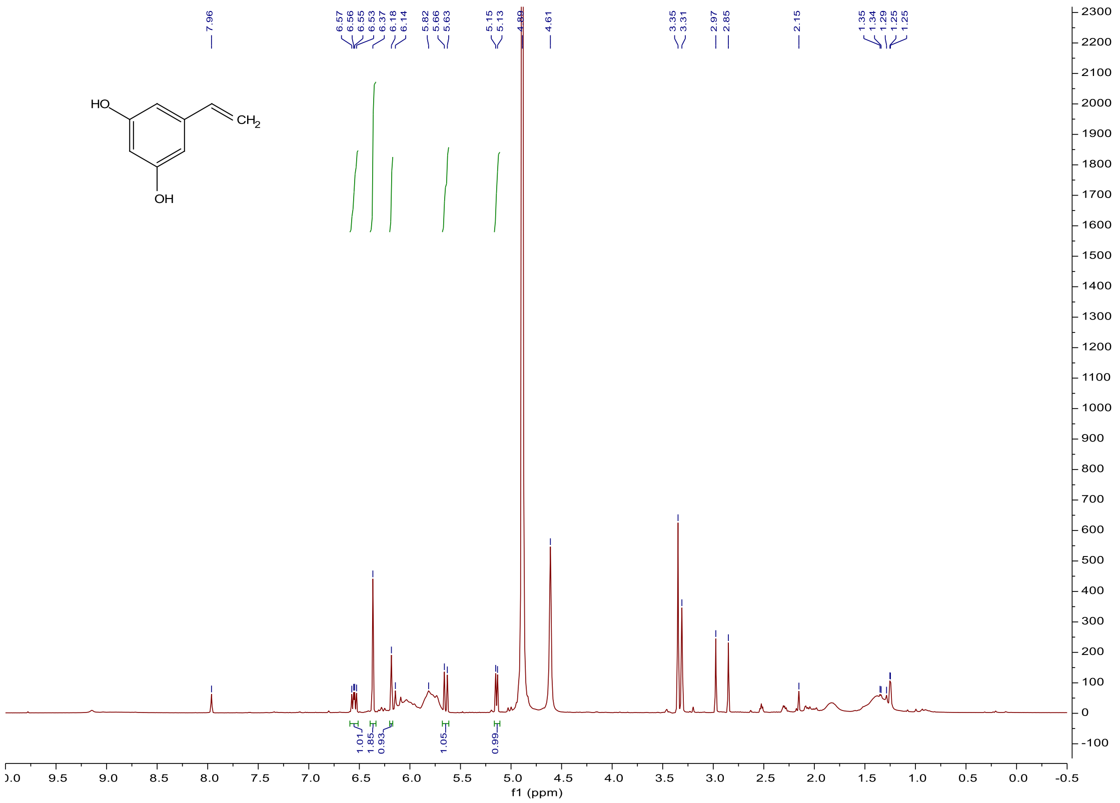

^{13}C-NMR (150MHz, CD_3OD-d_4) of compound 12

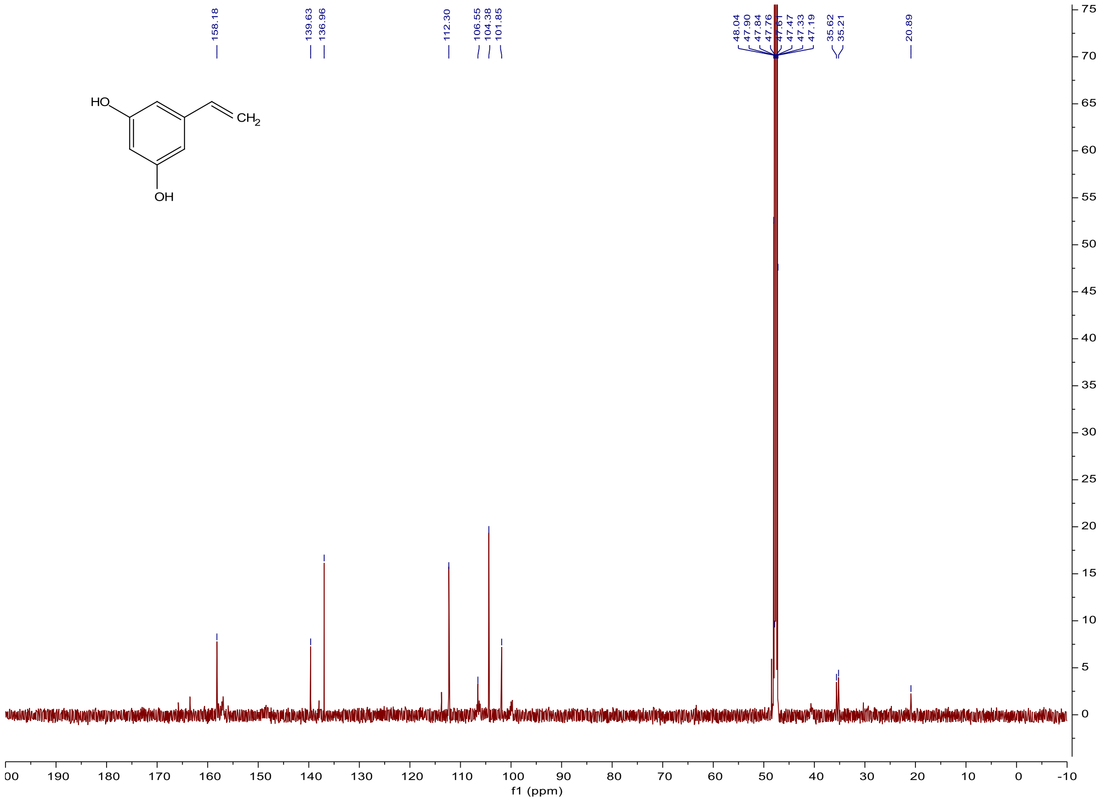

^{1}H-NMR (600MHz, $CDCl_3$) of compound 5

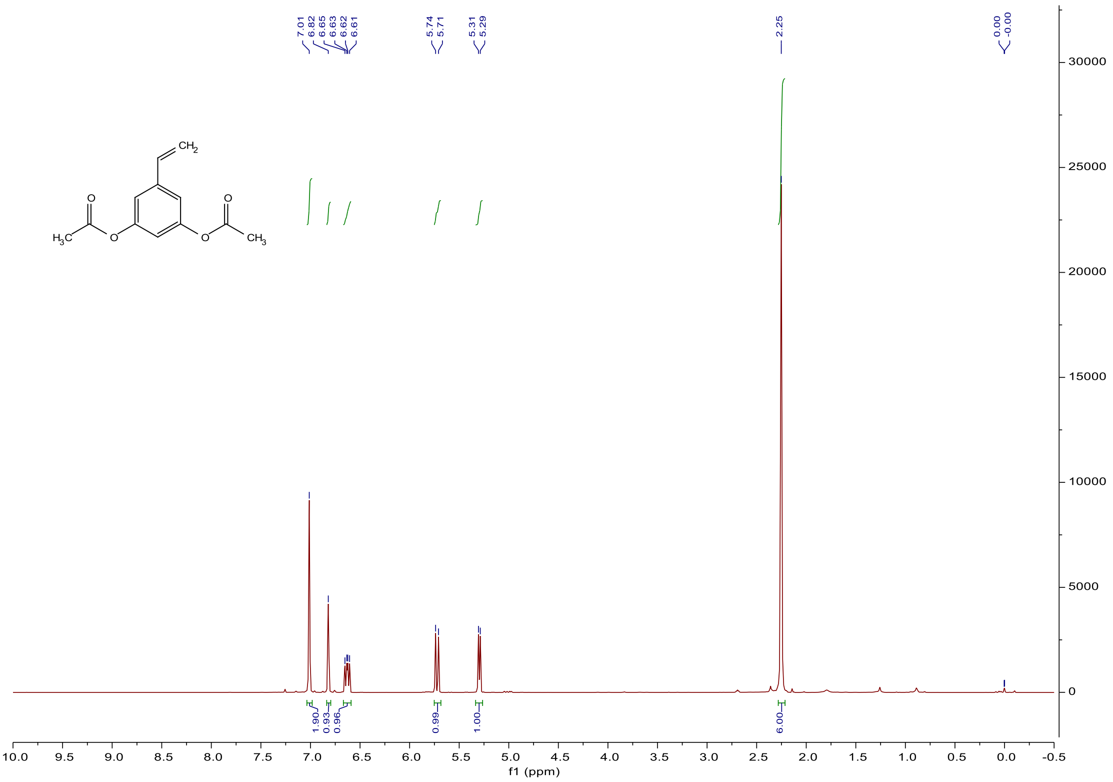

^{13}C-NMR (150MHz, $CDCl_3$) of compound 5

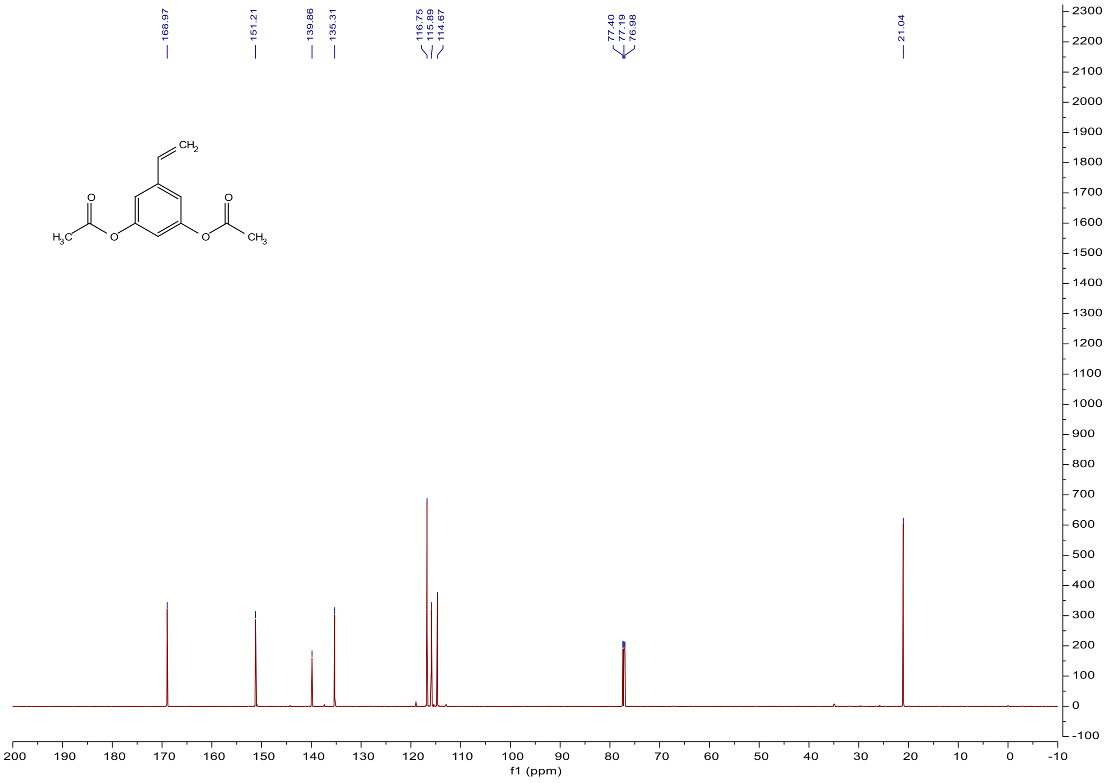

^{1}H-NMR (600MHz, $CDCl_3$) of compound 11

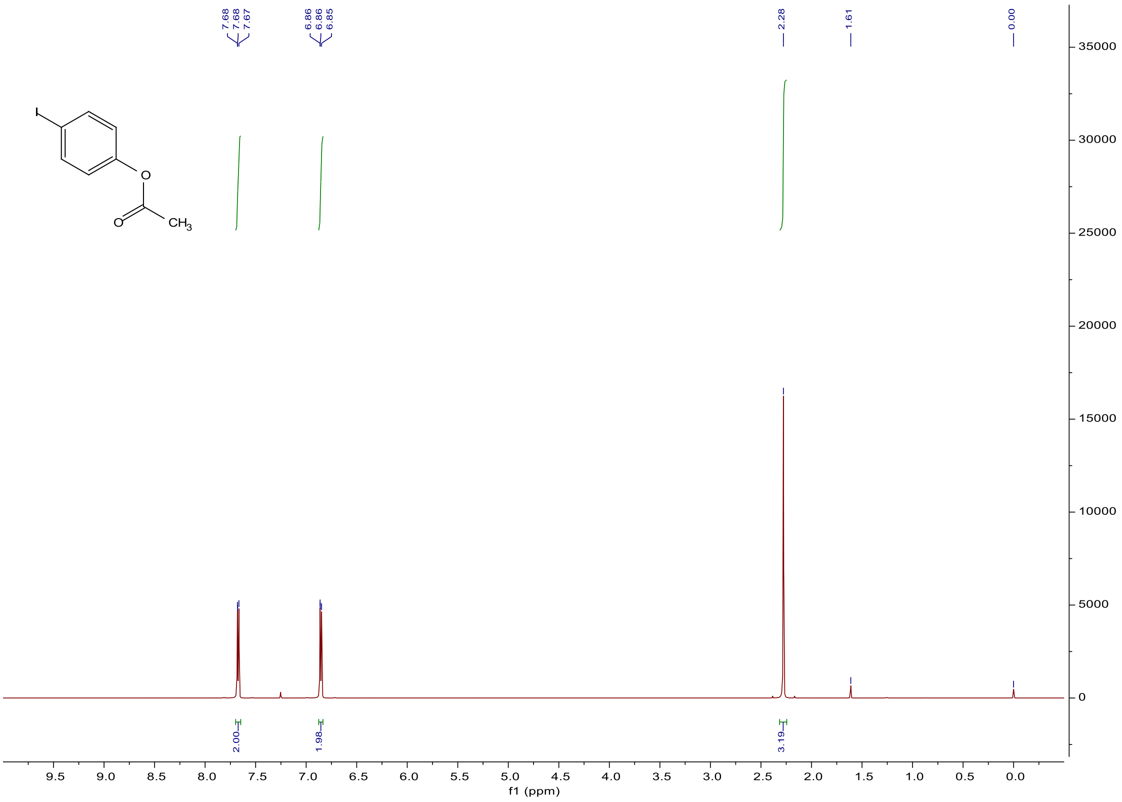

^{13}C-NMR (150MHz, $CDCl_3$) of compound 11

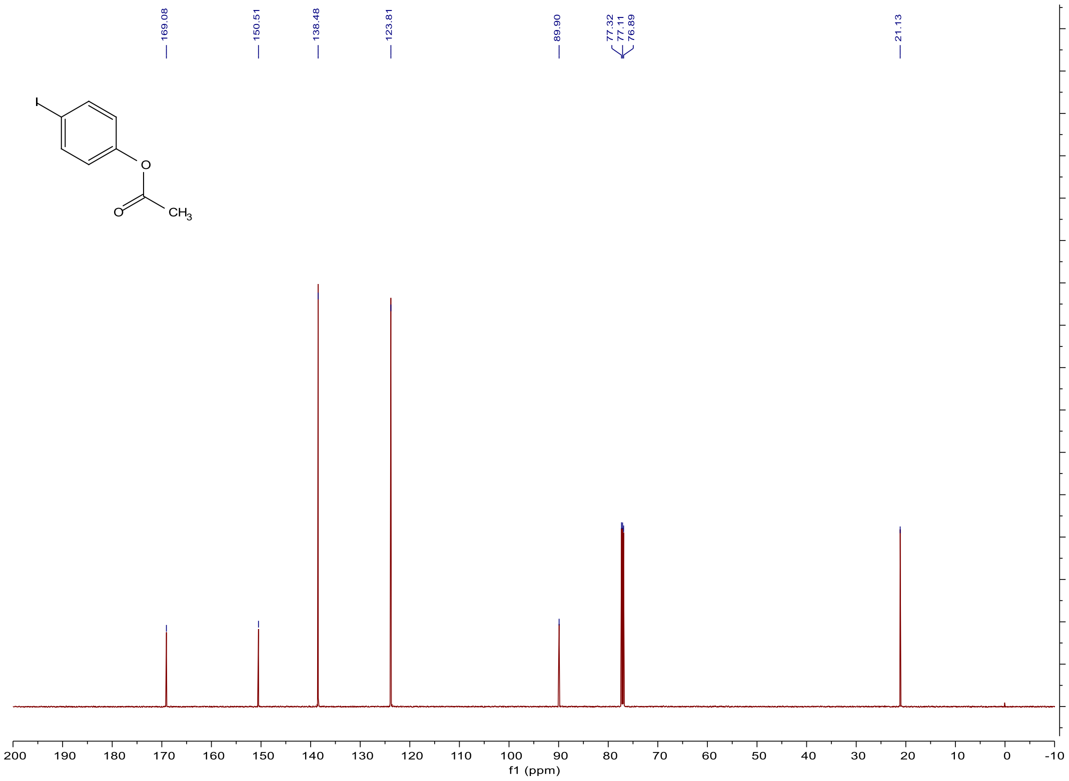

^{1}H-NMR (600MHz, $CDCl_3$) of compound 4

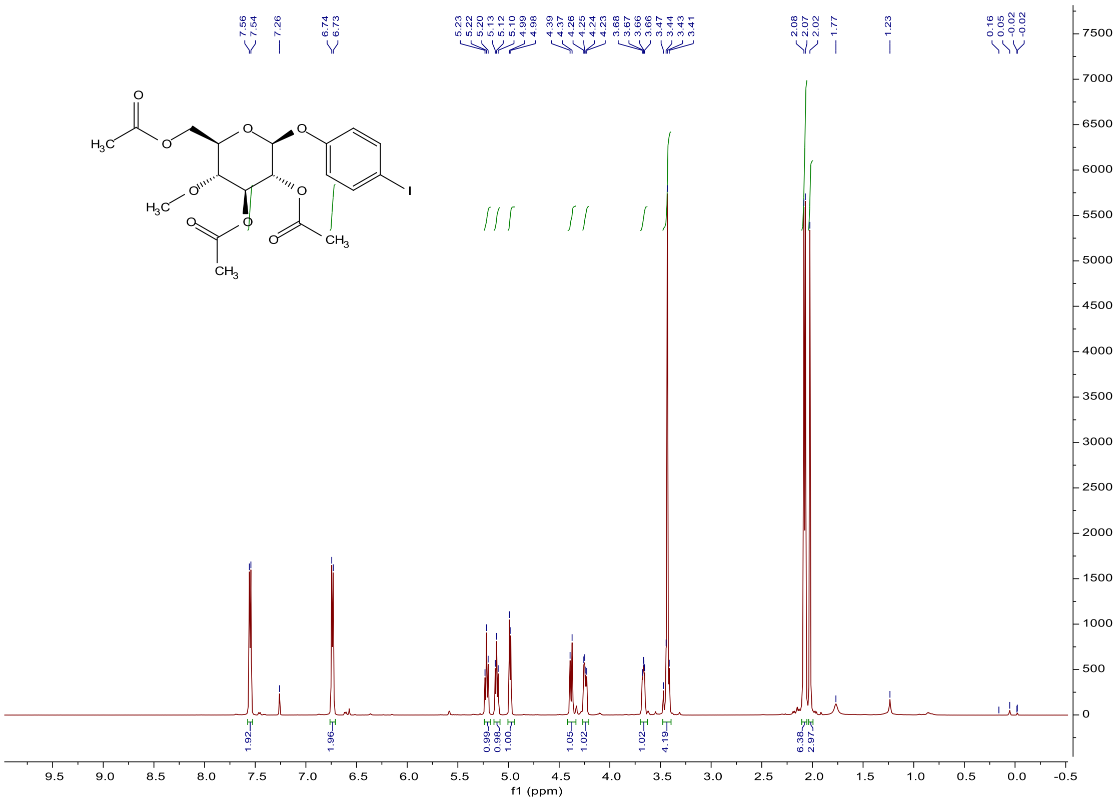

^{13}C-NMR (150MHz, $CDCl_3$) of compound 4

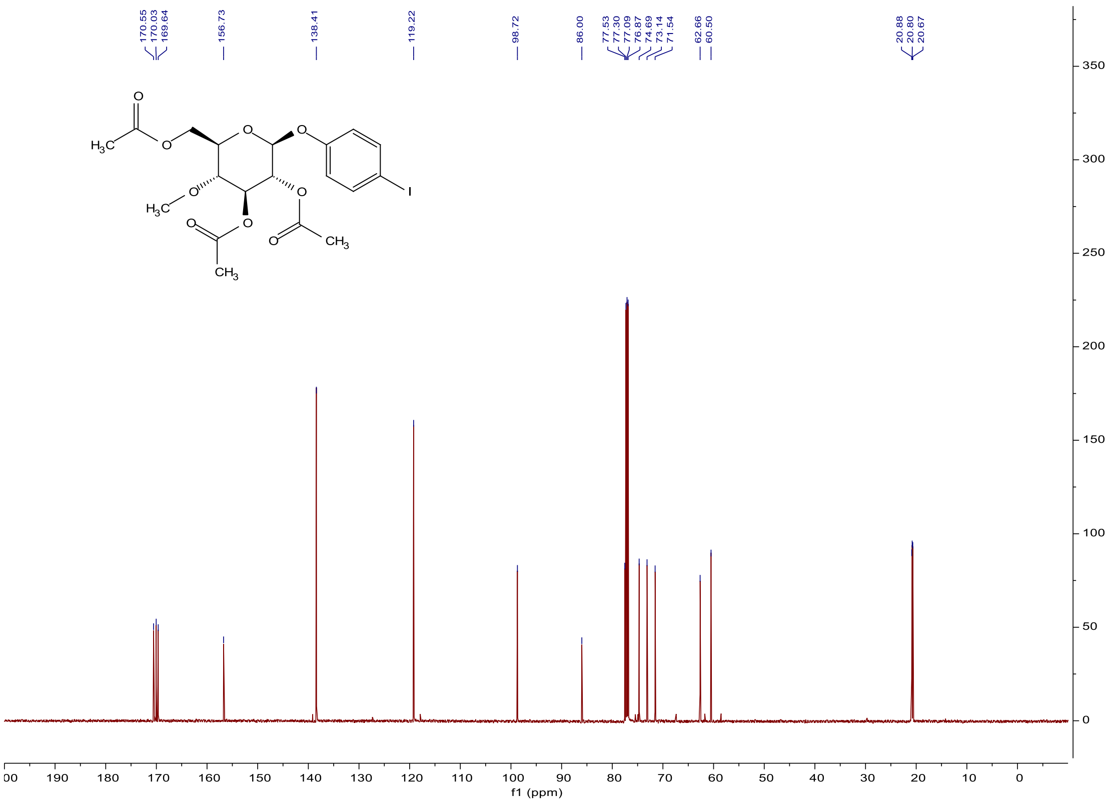

^{1}H-NMR (600MHz, $CDCl_3$) of compound 22

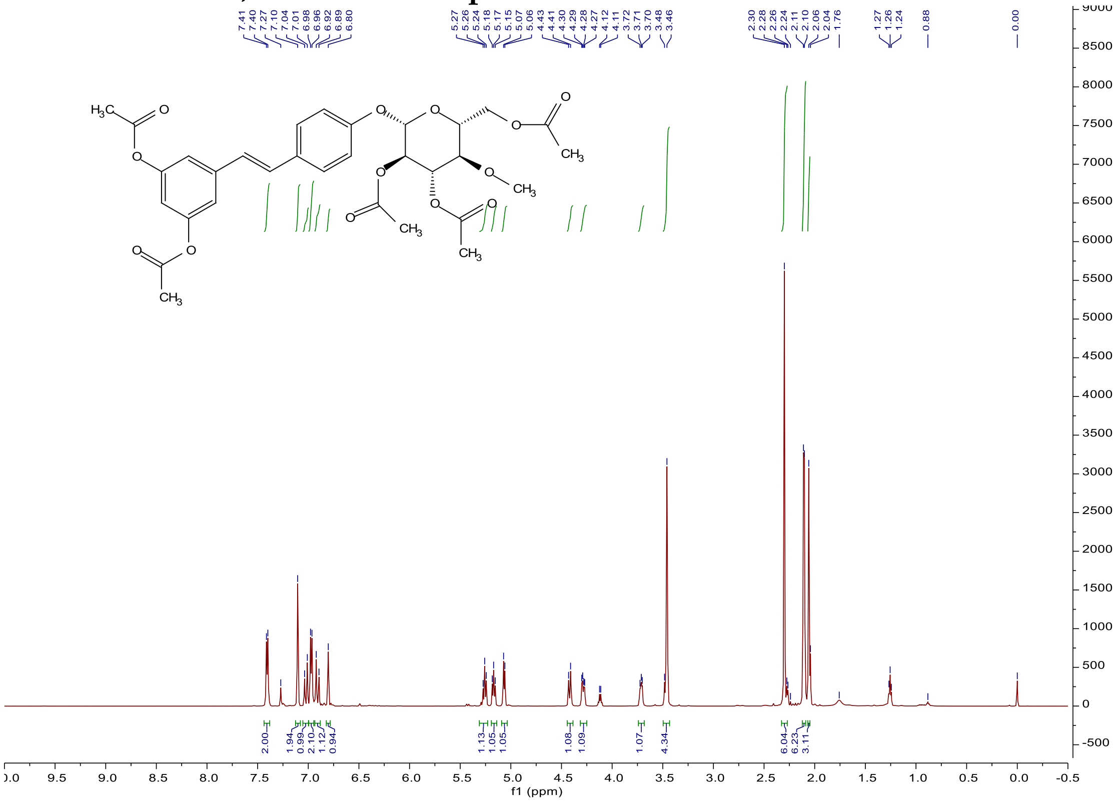

^{13}C-NMR (150 MHz, $CDCl_3$) of compound 22

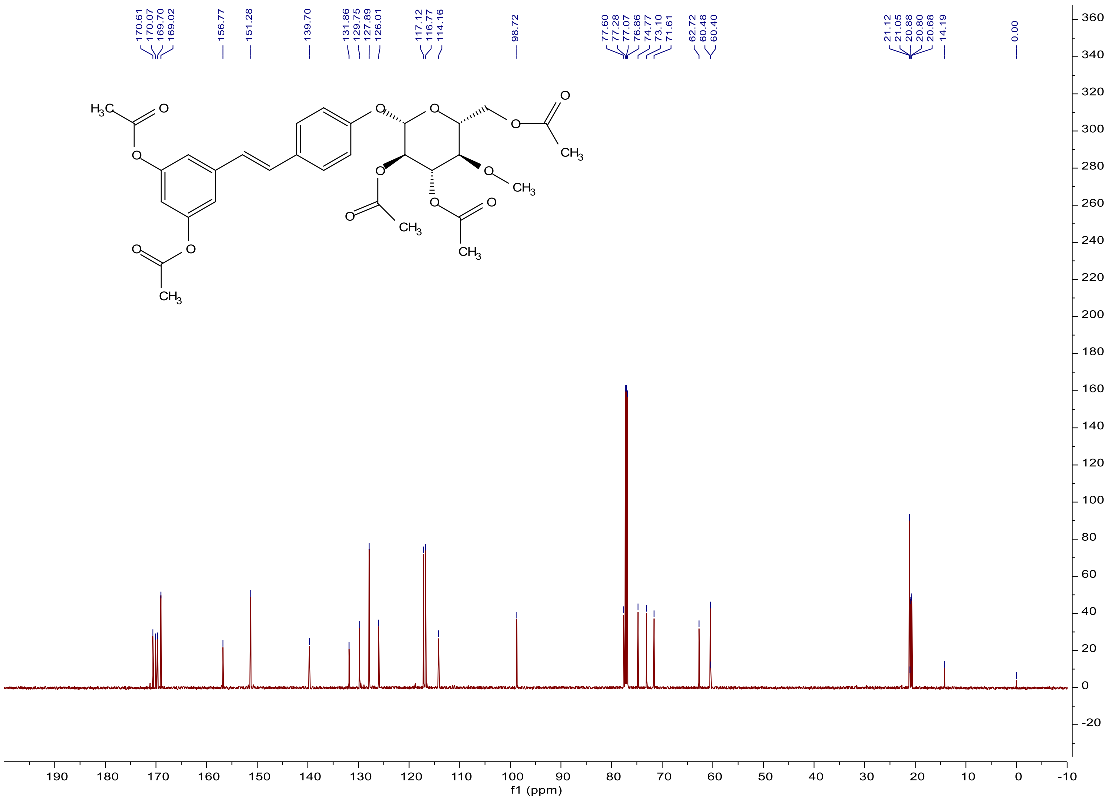

^{1}H-NMR (600MHz, $(CD_3)_2CO$)) of compound 2

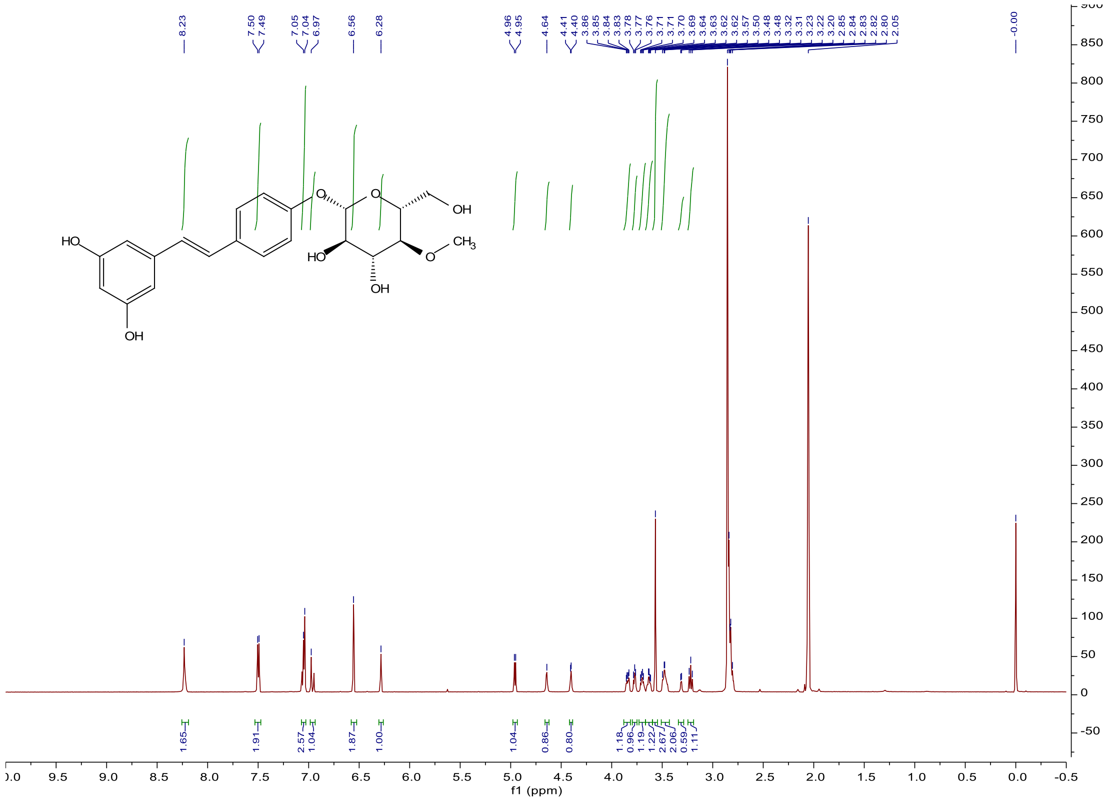

^{13}C-NMR (150MHz, $(CD_3)_2CO$) of compound 2

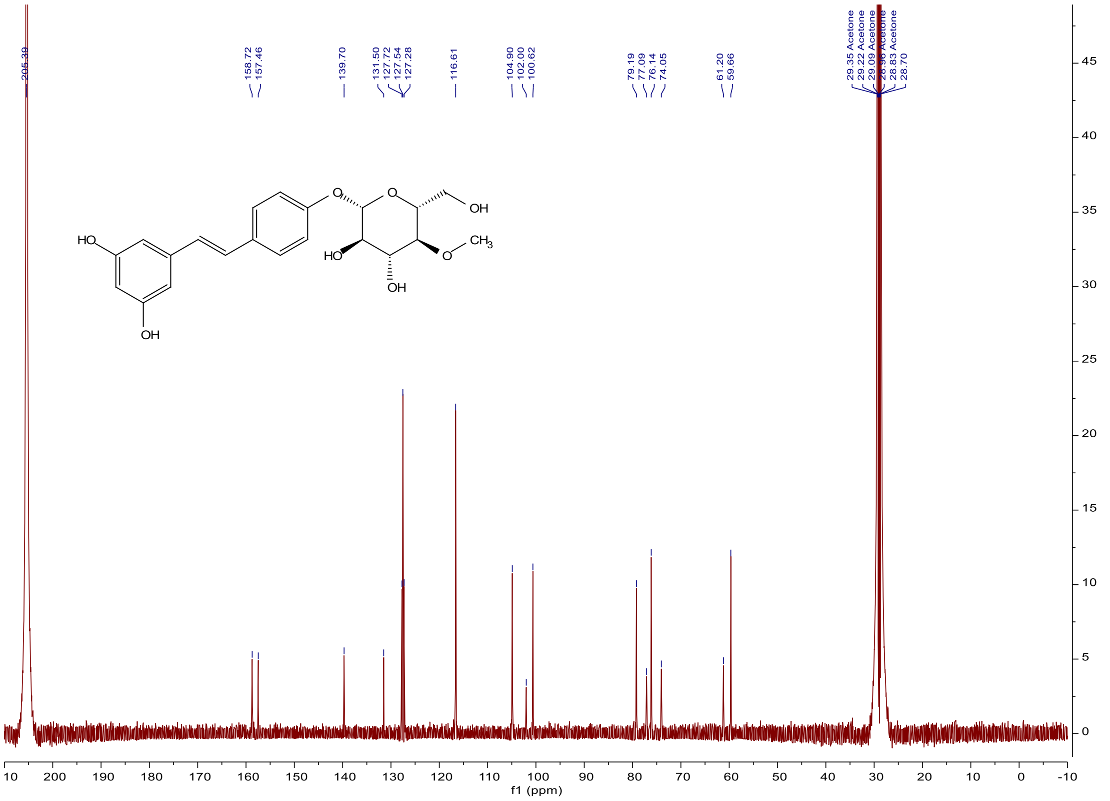

^{1}H-NMR (600MHz, CDCl3) of compound 10

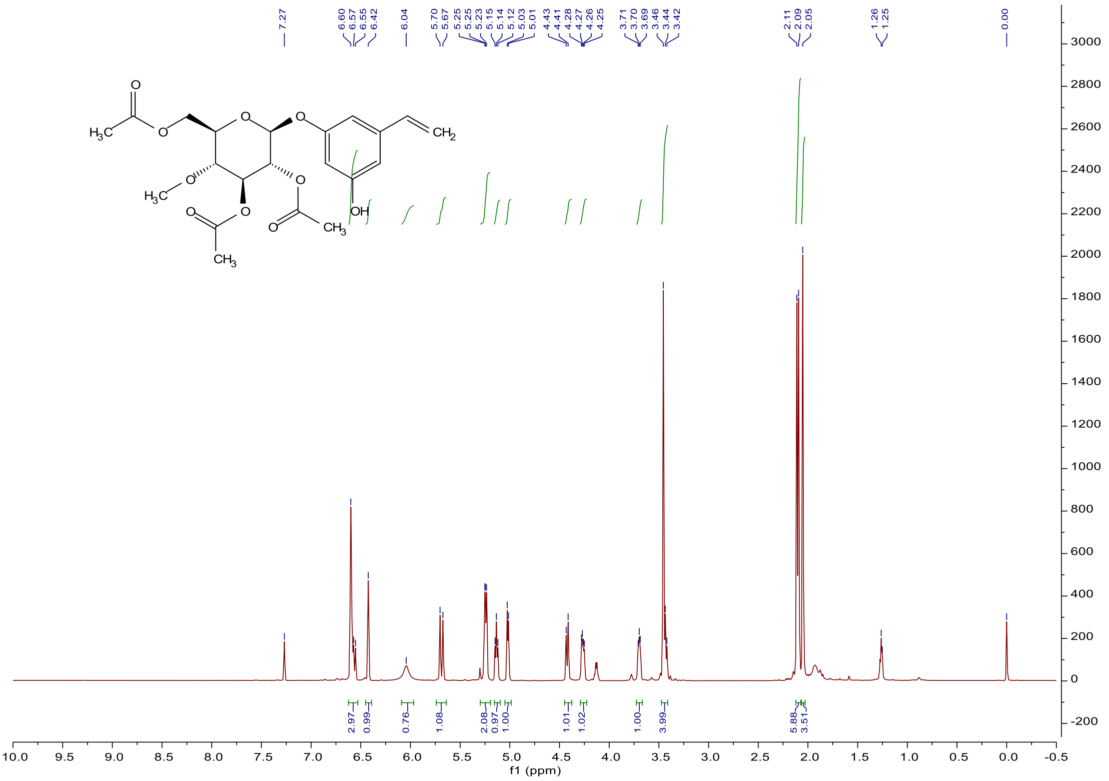

^{13}C-NMR (150MHz, CDCl3) of compound 10

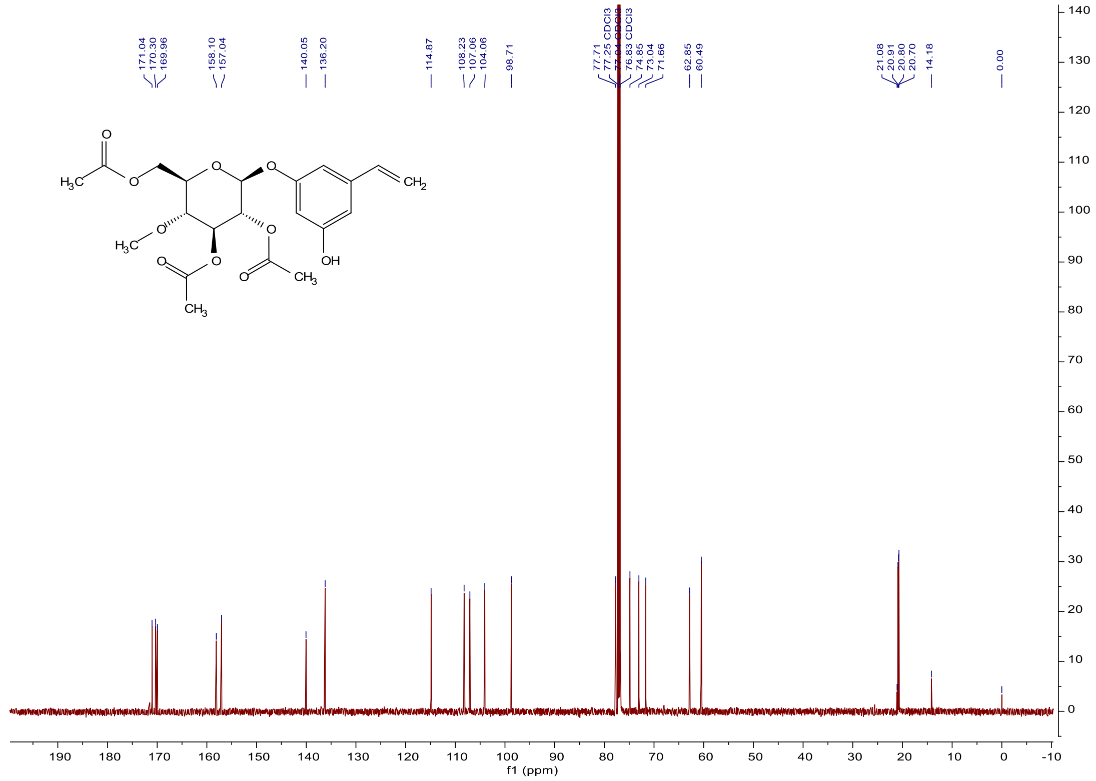

^{1}H-NMR (600 MHz, CDCl$_3$) of Compound 23

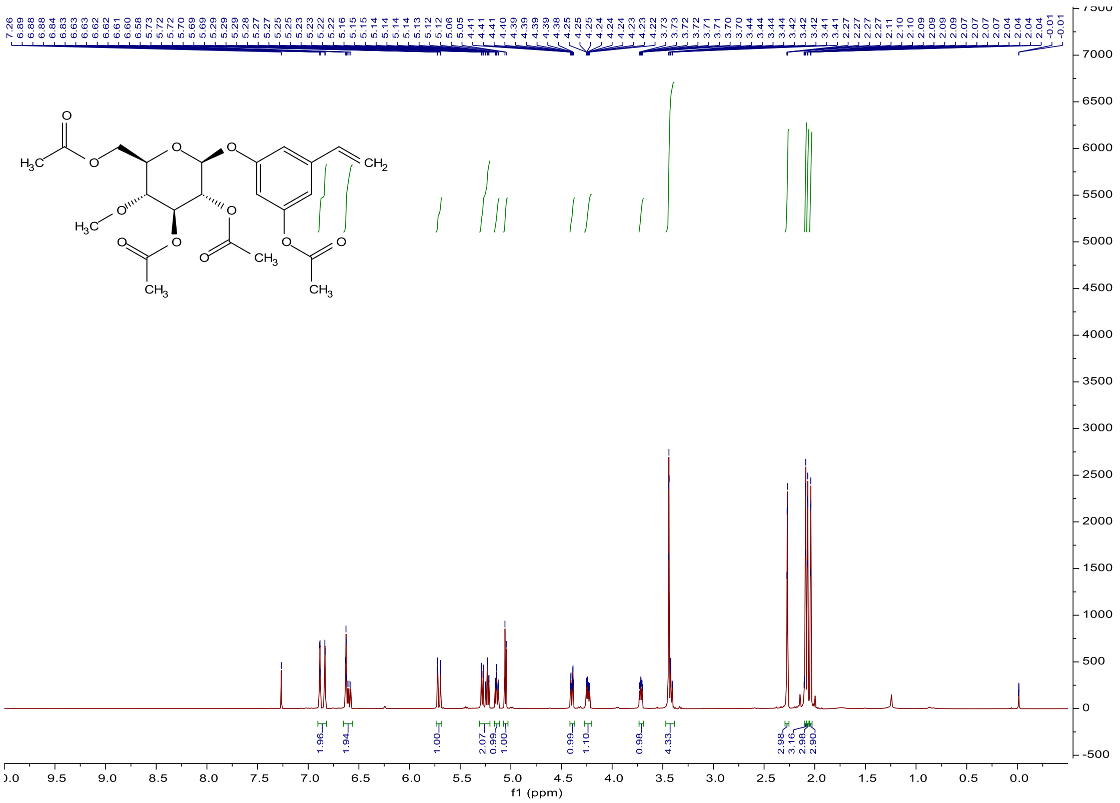

^{13}C-NMR (151 MHz, CDCl$_3$) of Compound 23

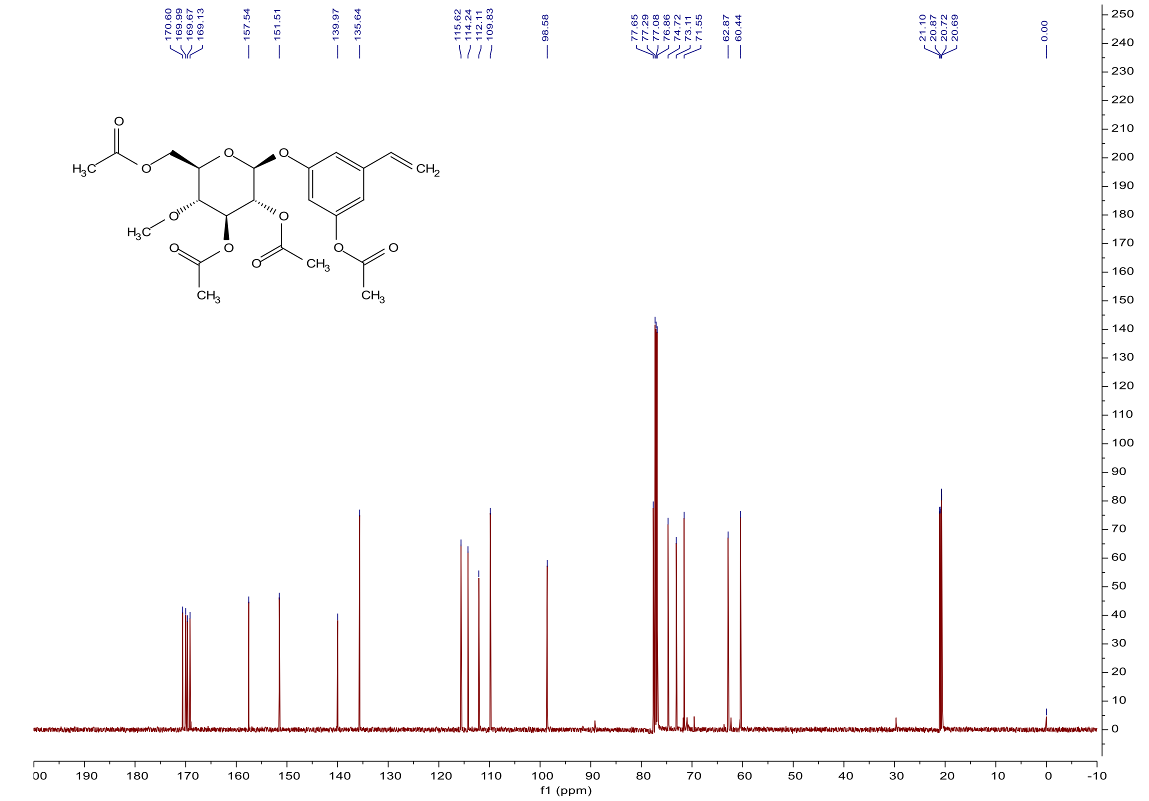

^{1}H-NMR (600MHz, $CDCl_3$) of compound 24

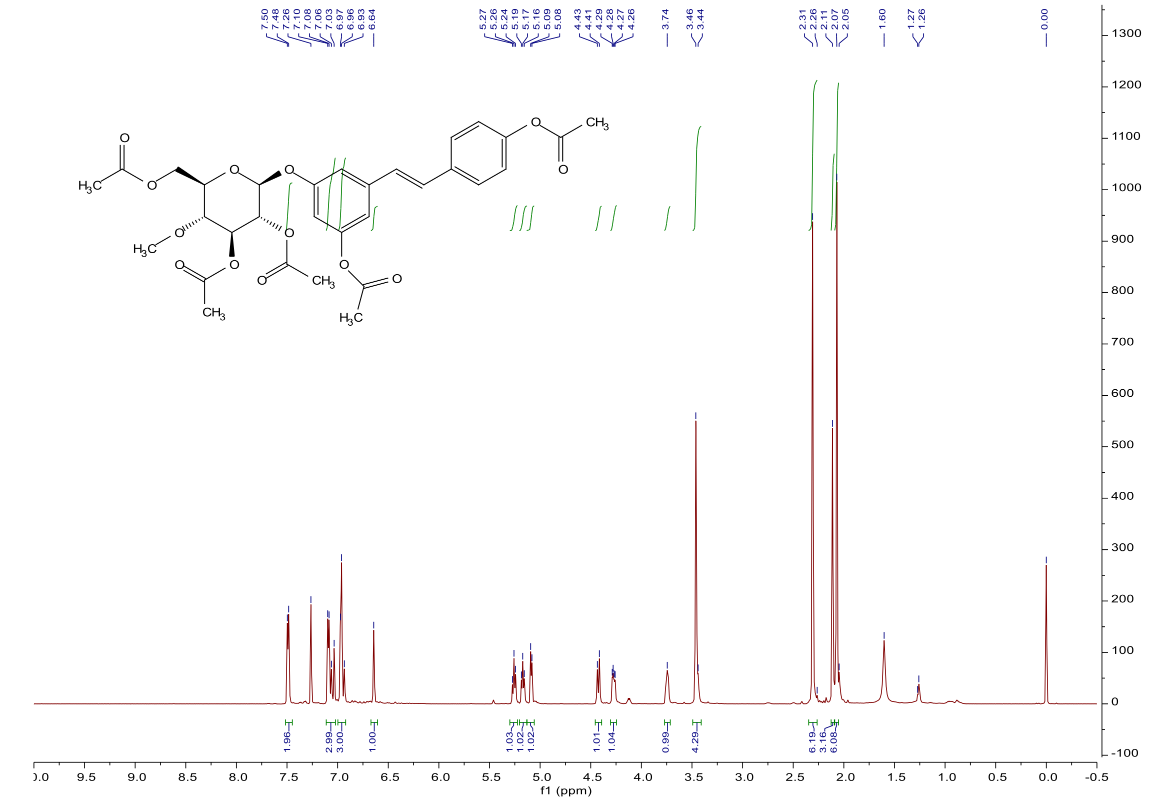

^{13}C-NMR (150 MHz, $CDCl_3$) of compound 24

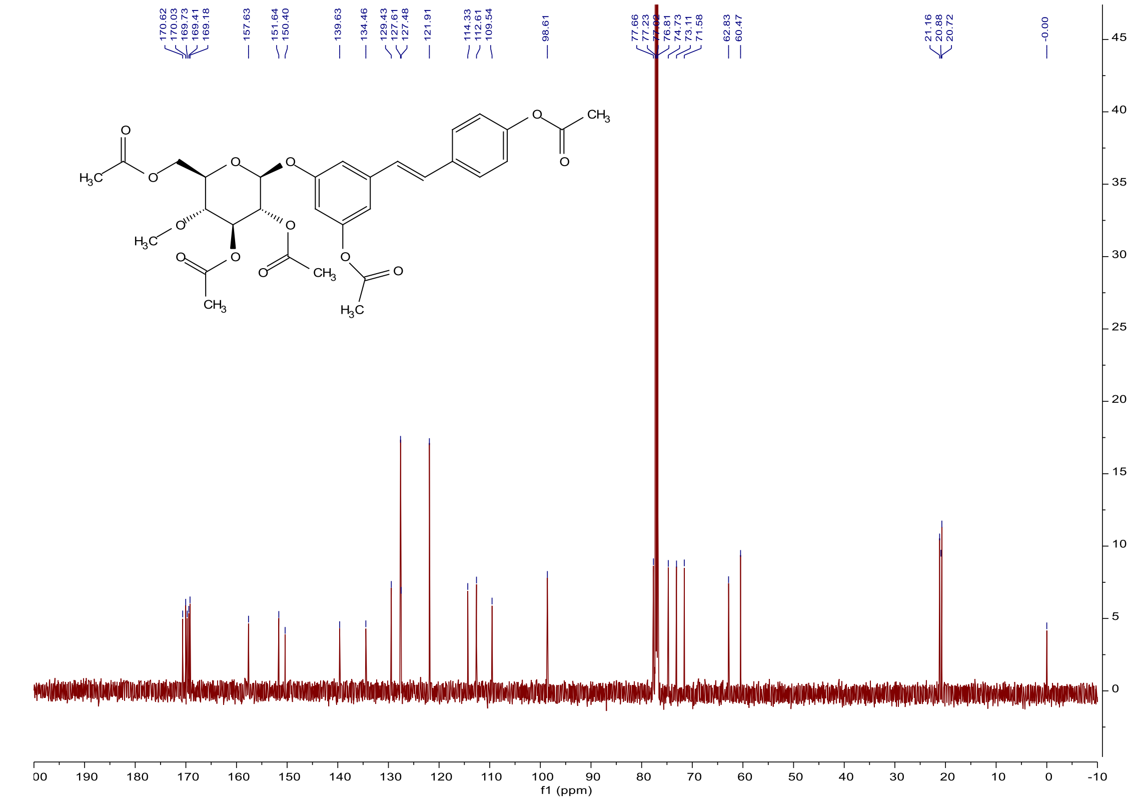

^{1}H-NMR (600MHz, $(CD_3)_2CO$) of compound 3

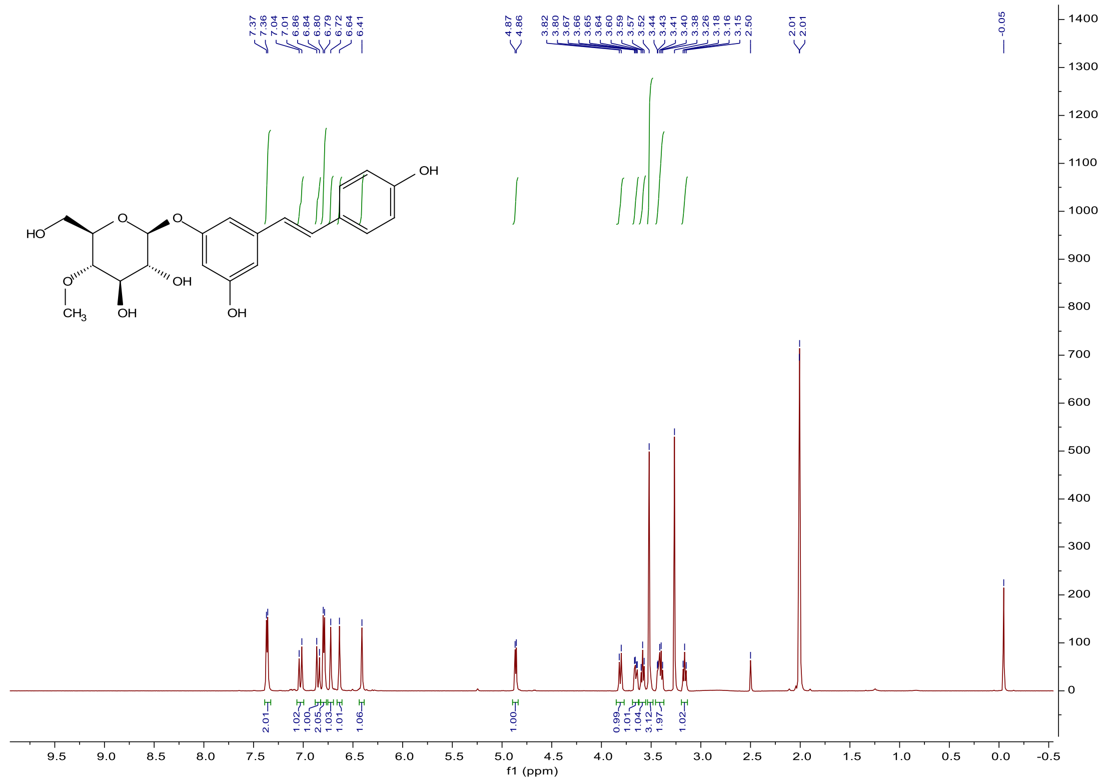

^{13}C-NMR (150 MHz, $(CD_3)_2CO$) of compound 3

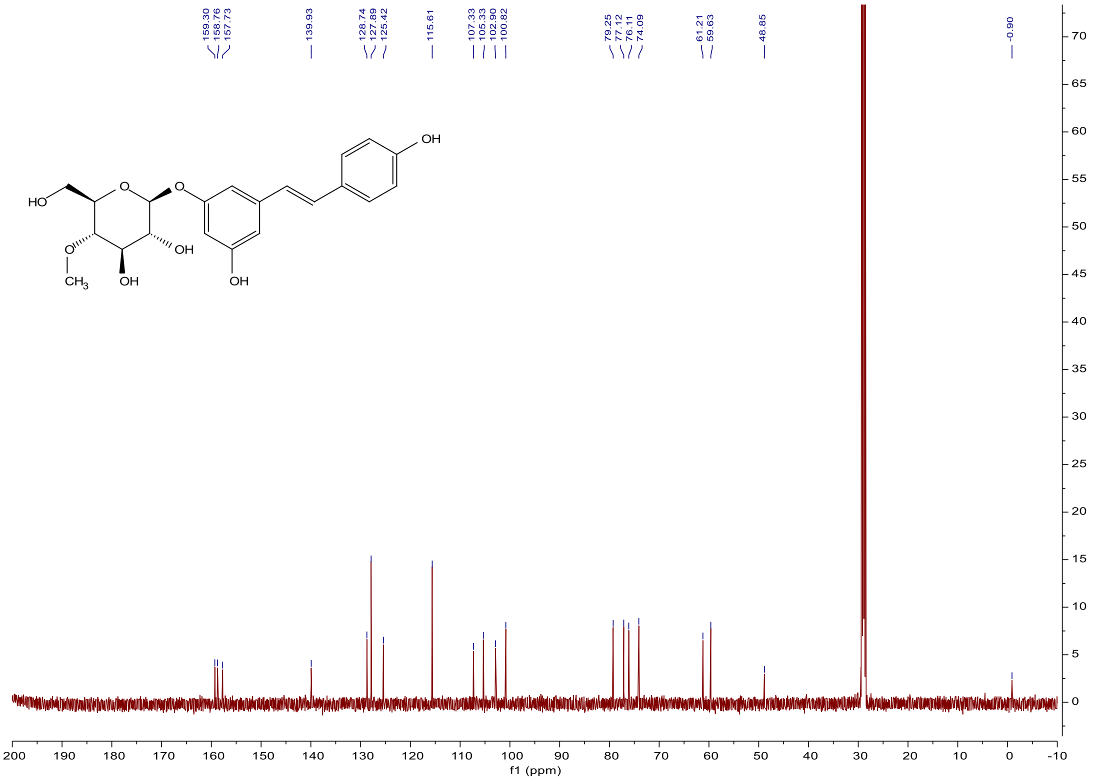
159.30
158.76
157.73
139.93
128.74
127.89
125.42
115.61
107.33
105.33
102.90
100.82
79.25
77.12
76.11
74.09
61.21
59.63
48.85
-0.90
OH
HO
O
O
OH
OH
O
CH3
OH
70
65
60
55
50
45
40
35
30
25
20
15
10
5
0
200
190
180
170
160
150
140
130
120
110
100
90
80
70
60
50
40
30
20
10
0
-10
f1 (ppm)
